# Introducing ART: A new method for testing auditory memory with circular reproduction tasks

**DOI:** 10.3758/s13428-024-02477-2

**Published:** 2024-09-09

**Authors:** Aytaç Karabay, Rob Nijenkamp, Anastasios Sarampalis, Daryl Fougnie

**Affiliations:** 1 Program in Psychology, New York University Abu Dhabi, Abu Dhabi, United Arab Emirates; 2 Center for Information Technology, University of Groningen, Groningen, The Netherlands; 3 Department of Psychology, Experimental Psychology, University of Groningen, Groningen, The Netherlands

**Keywords:** Auditory delayed estimation paradigm, Auditory working memory, Shepard tone complex

## Abstract

Theories of visual working memory have seen significant progress through the use of continuous reproduction tasks. However, these tasks have mainly focused on studying visual features, with limited examples existing in the auditory domain. Therefore, it is unknown to what extent newly developed memory models reflect domain-general limitations or are specific to the visual domain. To address this gap, we developed a novel methodology: the Auditory Reproduction Task (ART). This task utilizes Shepard tones, which create an infinite rising or falling tone illusion by dissecting pitch chroma and height, to create a 1–360° auditory circular space. In Experiment 1, we validated the perceptual circularity and uniformity of this auditory stimulus space. In Experiment 2, we demonstrated that auditory working memory shows similar set size effects to visual working memory—report error increased at a set size of 2 relative to 1, caused by swap errors. In Experiment 3, we tested the validity of ART by correlating reproduction errors with commonly used auditory and visual working memory tasks. Analyses revealed that ART errors were significantly correlated with performance in both auditory and visual working memory tasks, albeit with a stronger correlation observed with auditory working memory. While these experiments have only scratched the surface of the theoretical and computational constraints on auditory working memory, they provide a valuable proof of concept for ART. Further research with ART has the potential to deepen our understanding of auditory working memory, as well as to explore the extent to which existing models are tapping into domain-general constraints.

**Supplementary information:**

The online version contains supplementary material available at 10.3758/s13428-024-02477-2.

## Introduction

Theories about the nature of visual working memory have advanced considerably since the adoption of continuous reproduction tasks and mixture modeling frameworks. In continuous reproduction tasks (e.g., the delayed estimation paradigm), participants are asked to remember a feature value(s) of a stimulus (or set of stimuli) and to later reproduce this stimulus from a perceptually circular and uniform space (e.g., color; Prinzmetal et al., 1998; Wilken & Ma, 2004). In contrast to change detection and delayed report tasks (Jacobsen, 1936; Luck & Vogel, 1997), which provide discrete correct/incorrect responses, continuous reproduction tasks allow for graded memory responses. Commonly used circular continuous reproduction tasks have key benefits over dichotomous tasks (Skóra et al., 2021). These benefits include a more detailed perceptual or representational quality than dichotomous tasks, as they provide a raw measure of distance between the perceived and remembered stimulus; they also constitute a bias-free measure, since dichotomous tasks may involve more liberal or conservative reports (Macmillan & Creelman, 2004; Wilken & Ma, 2004). Critically, by utilizing a circular space for the report dimension, modeling frameworks are able to take a distribution of errors obtained from continuous reproduction tasks and isolate separate putative states which link to distinct cognitive mechanisms. The most well-known of these modeling frameworks is the standard mixture model (Zhang & Luck, 2008), which argued that a major constraint on visual memory was an upper limit on the number of items that could be stored. In this view, error distributions can be described as responses arising from the weighted mixture of two states: an “in-memory” state captured by a circular normal distribution with a width corresponding to the fuzziness of memory, and an “out-of-memory” state characterized by a uniform distribution over the possible values (since uninformed responses are random relative to the stimulus). In recent years, adaptations and revisions of this framework have led to many notable advancements in our understanding of visual working memory (for a systematic comparison of computational working memory models, see Van den Berg et al., 2014). While some theoretical frameworks reject the idea of separate memory states (e.g., Schurgin et al., 2020), the ability to construct and test generative models has led to rapid progress in our understanding.

A side effect of the focus on continuous reproduction tasks, however, has been a reduction in the scope of possible stimulus spaces in which working memory can be investigated. Continuous reproduction tasks have utilized color, orientation, location, motion, face, and shape spaces (e.g., Asplund et al., 2014; Bays & Husain, 2008; Karabay et al., 2022; Li et al., 2020; Wilken & Ma, 2004; Zokaei et al., 2011). Notably, these stimuli are all part of the visual domain. Prior to the development of mixture modeling techniques, a central question in the working memory literature was the degree to which working memory limits reflect a domain-independent limitation or whether separate limitations and properties exist for the different modalities such as vision and audition (e.g., Cowan, 1998; Fougnie & Marois, 2011; Morey et al., 2011; Saults & Cowan, 2007). Although there have been a few instances where continuous reproduction tasks were used to assess auditory working memory, the stimulus spaces utilized in these approaches lacked circularity (Clark et al., 2018; Hedger et al., 2018; Kumar et al., 2013; Lad et al., 2020, 2022) or uniformity (Joseph et al., 2015), and most of them are not openly accessible (but see Lad et al., 2022). Because of these limitations, existing paradigms have not been adopted more generally and research on auditory working memory has not fully benefited from the computational modeling frameworks developed in recent years. These frameworks often use error responses to separate responses into distinct mechanisms. For example, some responses may reflect noisy target memories while others may reflect random responses (Zhang & Luck, 2008). Circular spaces simplify the separation of putative response types. Further, uniform circular spaces reduce biases and distortions that can arise in a bounded space (e.g., responses that are biased toward the center of a bounded space, Thiele et al., 2011). Generating an auditory stimulus space that is both circular and uniform is not as intuitively obvious as when utilizing visual features. Unlike certain visual features that lend themselves naturally to a circular space, such as color and orientation, stimuli in auditory working memory tasks have often had discrete, noncontinuous values and have varied over a bounded rather than a circular space. This makes the interpretation of putative guess responses challenging. Therefore, it is paramount to identify a stimulus set that lends itself to use in a circular continuous space that is uniform in nature.

To address this gap in the literature, we have developed an auditory continuous reproduction task that utilizes a uniform circular space consisting of Shepard tones drawn from a Shepard scale (Shepard, 1964). Shepard tones are complex waveforms that are known to create the auditory illusion of a tone that is infinitely rising or falling in terms of its pitch. This illusion is created by simultaneously playing back different frequency components spaced at successive octave intervals of a tonal pitch (i.e., the frequency of each component above its lowest included octave is exactly twice the frequency of the component in the octave just below), where the amplitude for each frequency component is gradually tapered off to sub-threshold levels for components that are further away from the specified fundamental frequency. For a circular reproduction task utilizing a stimulus space composed of 360 distinct Shepard tones that are logarithmically spaced in a single octave in terms of their fundamental frequencies, it is essential that the stimulus space “wraps around” a circle. Shepard tones allow for this circular wrapping, as they allow for differentiation in pitch class or pitch chroma (i.e., the specific note that is played) but are ambiguous in terms of their pitch height (i.e., the specific octave the note played is in; Deutsch, 1984; Deutsch et al., 2008; Shepard, 1964; Siedenburg et al., 2023). As a result, the transition from the higher end of the fundamental frequency range of the octave making up the Shepard scale (i.e., 360°) back to the lower end of the same octave (i.e., 1°) is imperceptible, and instead the illusion of a tone with a continuously falling or rising pitch is achieved.

The goal of this research is to provide (and make easily accessible) a proof-of-concept continuous reproduction task for auditory working memory based on Shepard tone space. Experiment 1 aims to test whether Shepard tone space meets the requirements for the Auditory Reproduction Task (ART), namely, perceptual circularity and uniformity. It is hypothesized that Shepard tone space will show perfect circularity and a uniform distribution (Deutsch, 1984; Deutsch et al., 2008; Shepard, 1964; Siedenburg et al., 2023). In Experiment 2, we introduce ART and manipulated set size that utilizes the circular Shepard tone space described above. We ask whether auditory working memory exhibits similar patterns to visual working memory, such as escalated reproduction errors with an increase in memory set size. We apply commonly used computational working memory models (e.g., standard mixture model, Zhang & Luck, 2008) to test underlying causes of error differences between set sizes. Moreover, we test task performance over time, assessing whether reproductions exhibit increasing precision or reveal variability with successive blocks. Next, in Experiment 3, we test the validity of ART by means of correlation with existing auditory and visual working memory paradigms. Finally, we discuss applications of ART and how it can shed light on the theoretical and computational constraints of auditory working memory, utilizing the same frameworks that have advanced our understanding of visual working memory.

## Experiment 1: Validating the circularity of Shepard tones

The Shepard tone was devised to dissect the specific contributions of chroma by making the pitch height ambiguous. By increasing the chroma of Shepard tones in a sequential manner, a cyclic pattern is established where a complete octave shift is perceptually (acoustically) identical to no shift at all. This generates the widely recognized Shepard scale illusion, which creates the perceptual experience of a continuous rise or fall in pitch. The evidence that Shepard tones make up a circular perceptual space comes from direction discrimination paradigms. In such a paradigm, responses are often divided equally between perceived upward and downward shifts for the same Shepard tone when compared to another tone that is separated by half an octave (Deutsch, 1984; Deutsch et al., 2008; Shepard, 1964; Siedenburg et al., 2023). Although the perception of the Shepard tone space was found to be circular, the literature has not quantitively assessed its circularity. By using a novel *circularity* value, we quantitatively assessed the circularity of the Shepard tone space (Li et al., 2020). Further, we qualitatively investigated the uniformity of the distribution of the tones. To test these questions, we generated 15 discrete Shepard tones with fundamental frequencies making up a single octave range and asked participants to rate the similarity of the tones played. We tested the circularity of this perceptual space by applying multidimensional scaling on similarity judgments, which is done by analyzing the similarity ratings of tone pairs and then placing those stimuli in a two-dimensional space based on their perceived similarity (Shepard, 1980). Additionally, we qualitatively examined the distances of the tones relative to each other to further assess their uniformity.

### Methods

#### Participants

The number of participants to assess circularity of Shepard tone space was determined by following Li et al.’s sample size (2020). As we use Bayesian tests for statistical analyses, we did not estimate sample sizes in advance, as Bayesian tests do not require sample size calculation (Berger & Wolpert, 1988). Participants who did not have an average similarity rating of 4 or higher on identical sound samples (e.g., tone 1 vs. tone 1) during the similarity rating phase of the experiment were excluded from further data analysis. This exclusion criterion was applied because it indicated that these participants could not sufficiently discriminate tone pairs. In total, 28 individuals participated in Experiment 1, four of which were excluded from the study due to this exclusion criterion. The final sample consisted of 24 students (11 female, 13 male) from the New York University Abu Dhabi (mean age = 20.3 years, ranging from 18 to 24 years). All participants reported having normal hearing. Participants were assigned to one of two groups depending on their participant number parity, with 12 participants assigned to group 1 and 12 participants assigned to group 2. Each of these two groups rated the subjective similarity of different stimuli sets. Participants were compensated for their participation in the experiment with vouchers worth 50 dirhams. The study was approved by the ethical committee of the New York University Abu Dhabi Psychology Department. Informed consent forms were collected prior to data collection, and the research was conducted in accordance with the Declaration of Helsinki (2008).

#### Apparatus

OpenSesame 3.3.12 (Mathôt et al., 2012) using the Expyriment back-end (Krause & Lindemann, 2014) was used for trial preparation and data collection under the Microsoft Windows 10 operating system. Participants were individually seated in sound-attenuated testing cabins about 60 cm from a 24-inch BenQ XL2411 screen. The screen resolution was set to 1024 by 768 pixels. The stimulus sounds were played through Audio-Technica ATH-M50x over-ear headphones. The playback device’s volume level was fixed at 20% of the maximum, to a comfortable, audible level. Responses were collected using a standard computer mouse.

#### Stimuli

The auditory stimulus space used in this experiment consisted of a Shepard tone complex (STC; Shepard, 1964). The use of the STC space proved essential for mapping the fundamental frequency of the played-back Shepard tones to a circular perceptual task, as these kinds of tone complexes are known for enabling differentiation in pitch class or pitch chroma (i.e., the specific note played) while being ambiguous in pitch height (i.e., the specific octave a note is in). The *ShepardTC* function (Böckmann-Barthel, 2017) was used in MATLAB to generate the tones used as the auditory stimulus space. Each Shepard tone is composed of different frequency components spaced at successive octave intervals of a tonal pitch within 30 and 16,000 Hz, where the amplitude for each frequency component that is further away from the specified fundamental frequency is gradually tapered off following a cos^2^ bell-shaped spectral envelope until sub-threshold levels are reached. Note that this envelope procedure differs slightly from that used in the original work of Shepard (1964), where the spectral envelope was applied to the sound levels of the different frequency components rather than the amplitude. The fundamental frequencies of the tones making up the auditory stimulus space were in a single octave ranging from 278.4375 Hz to 556.875 Hz (i.e., the same pitch chroma one full octave higher). The subharmonic frequencies included a range of nine octaves between 30 and 16,000 Hz (i.e., 31.25, 62.5, 125, 250, 500, 1000, 2000, 4000 and 8000 Hz were used to create a Shepard tone with a fundamental frequency of 500 Hz). The tones were generated using a sampling frequency of 44,100 Hz. The STC space was logarithmically transformed using a base-2 (log_2_) scale to a 1–360° circular space, such that 1° was equivalent to 279.2109 Hz and 360° was equal to 556.8750 Hz. Each increase of 1° was equal to an increase in the fundamental frequency by a factor of $\sqrt[360]{2}$. Fifteen discrete tones with a 24° difference were used for assessing the circularity of the perceptual space of the tones (Table 1).

**Table 1:** STC space per participant set. The fundamental frequency (in Hz) of the tones and their corresponding angles in a 360° circular space are listed. Tones with asterisks are anchor tones

Participant set 1		Participant set 2	
STC (Fund. Hz)	STC (°)	STC (Fund. Hz)	STC (°)
304.81	48	291.05	24
334.32	96	319.23	72
350.13*	120*	350.13*	120*
384.04	168	366.69	144
421.22	216	402.20	192
441.14*	240*	441.14*	240*
483.86	288	462	264
530.70	336	506.74	312
555.80*	360*	555.80*	360*

#### Procedure

The nature of Shepard tones allows an octave tone space to psychologically “wrap” from the end of the space to the beginning. To demonstrate this, we adopted the procedure from Li et al. (2020) to assess the circularity of our auditory space. There were a total of 163 trials, of which 10 were practice trials. Each trial had two phases: the initial judgment phase and the similarity rating phase. The experiment took approximately 60 min to complete per participant.

##### Initial judgment phase

**Fig. 1:**
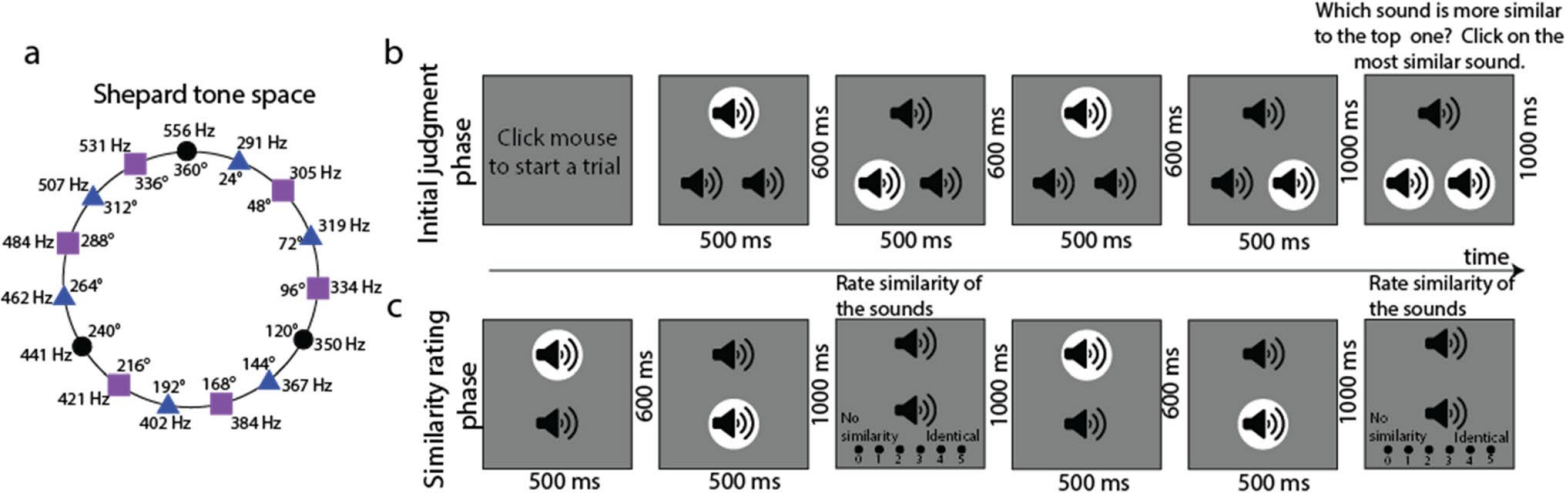
STC space and task demonstration. **a** The STC space and corresponding locations on a 360° space. Black dots on the sound circle show the anchor tones that were played to both participant groups. Blue triangles are tones used in participant group 1, and purple squares are tones used in participant group 2. **b** Initial judgment phase of the experiment. White disks appeared on the tone that was playing. Participants were asked to click the most similar bottom sound relative to the top sound. **c** Similarity rating phase. Tone pairs in the initial judgment phase were played sequentially in random order (e.g., top and left followed by top and right). Participants reported the subjective similarity of the sound samples, choosing from a Likert-type scale ranging from 0 to 5

Every trial started with a mouse click. After the mouse click, three speaker icons appeared on the screen, forming a triad (Fig. 1b). First, 600 ms after the icons appeared, the tone at the top location played for 500 ms, followed by one of the two tones at the bottom locations for the same duration. Later, the tone at the top location played again for 500 ms. Finally, the tone at the remaining bottom location played for the same duration. A white circle appeared behind a speaker icon while the corresponding sound was being played. The order in which the bottom left and right sides of the triad were played was randomized and distributed equally across trials (the left tone played first in 76 trials and the right tone played first in 77 trials, or vice versa). Each tone was separated by a 600-ms inter-stimulus interval (ISI). After a 1000-ms interval after the offset of the last tone, the response screen appeared. Participants were then asked to click on one of the sounds at the two bottom locations that they perceived to be most like the sound corresponding to the top location. Data from the initial judgment phase were not used in any analyses; the purpose of this phase was to calibrate participants’ pairwise similarity ratings for the following similarity rating phase.

##### Similarity rating phase

After a one-second inter-phase interval, a screen with two sound icons appeared, with one of them located at the top of the screen and the other located at the bottom of the screen (Fig. 1c). The top tone was identical to the tone that was presented in the same location during the initial judgment phase. The bottom tones corresponded to the tones located on either the bottom left or right side of the triad from the previous initial judgment phase. The order of the tone pairs corresponding to the bottom location was randomized and distributed equally across trials (the left tone played first in 76 trials and the right tone played first in 77 trials, or vice versa). First, the top tone was played for 500 ms, followed by the first bottom tone that was played for the same duration with a 600-ms ISI between the tones. A 1000-ms interval was inserted between the offset of the bottom tone and the response screen. Participants were then asked to judge the similarity of the two tones using a Likert scale ranging from 0 (no similarity) to 5 (identical). After an interval of 1000 ms after participants gave their response, the same response procedure was repeated for the remaining bottom tone that was also played in the initial judgment phase. Participants were instructed to rate the similarity of the tones using the whole range of the scale (Supplementary material Fig. S1 shows that participants were able to follow this instruction successfully).

#### Design

Each participant group listened to nine different tones (triangles and rectangles in Fig. 1a), three of which were identical in both groups (black dots in Fig. 1a). The tones that both groups listened to were used as anchor points to combine the perceptual spaces of the two groups during analysis. These anchor point tones were separated by a 120° difference on the circular auditory space (see Table 1 for the tones that were used in each participant set). Three tones were pseudo-randomly sampled in every trial during the initial judgment phase, resulting in all possible combinations of tones being sampled per participant. Each tone was compared with other tones a total of eight times (four out of eight times, one of the tones in each pair was shown at the top location of the triad during the initial judgment phase). Each tone was compared with itself twice during the experiment.

#### Assessing circularity

**Fig. 2:**
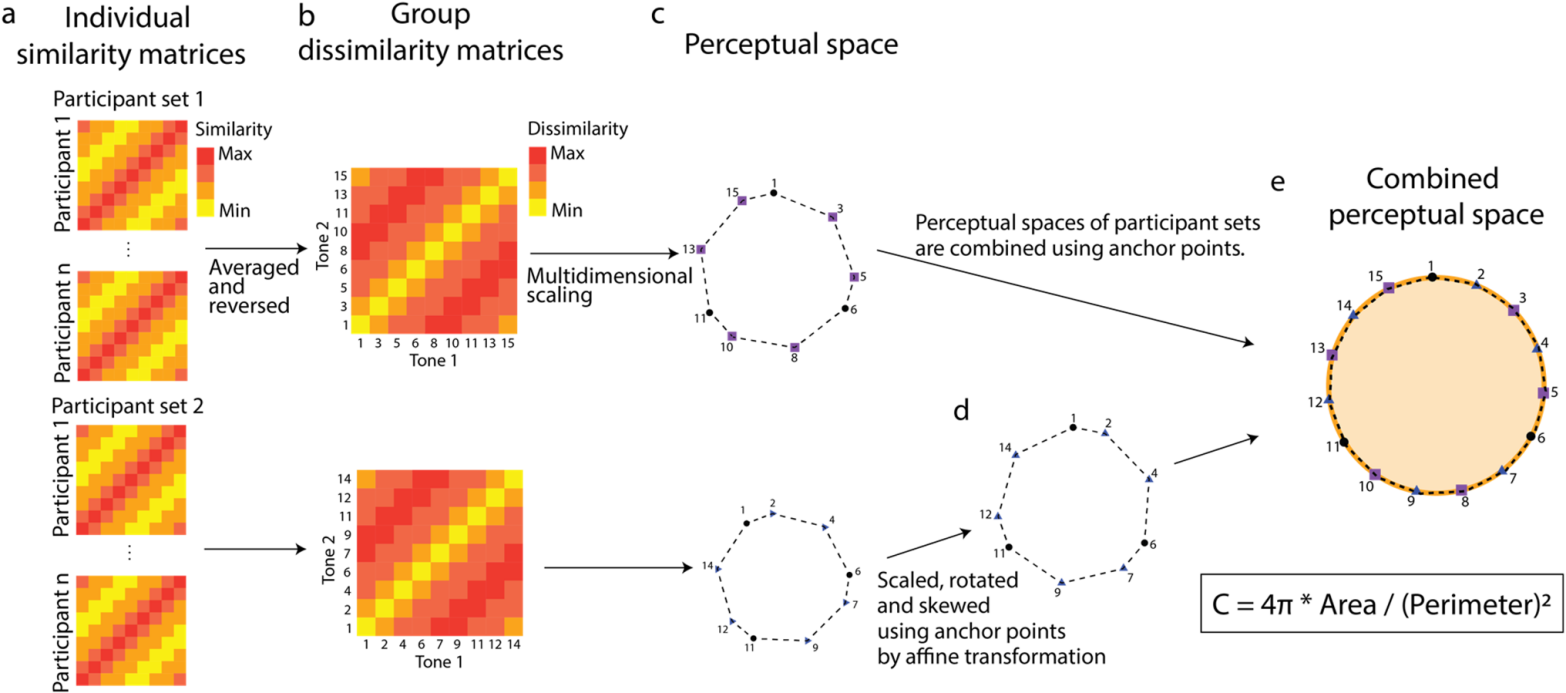
Illustration of the circularity analysis with fictional data. **a** Similarity matrix of each participant. **b** Group dissimilarity matrix created by averaging each group’s similarity matrix. After averaging, similarity ratings were reverse-coded. **c** Perceptual space of STC in each participant group. Shapes were created with multidimensional scaling of dissimilarity matrices per participant set. **d** Perceptual space of the second participant set after an affine transformation. **e** Perceptual tone space of the combined participant set. The circularity value was calculated on a splined and dashed line. Figure 2 is adapted from Li et al., (2020; Fig. 3) with permission from the American Psychological Association

We assessed the circularity of the STC space by calculating the circularity value (*C*) of the perceptual space based on multidimensional scaling (Li et al., 2020), which is the ratio of the area to the perimeter of the STC space, providing a quantitative measure of its circularity. First, similarity matrices using the pairwise similarity ratings of tones were created per participant. Pairwise similarity ratings were averaged regardless of their presentation order, creating a symmetrical similarity matrix for each participant (Fig. 2a). Next, we averaged each participant’s similarity matrix and reverse-coded them to create a single dissimilarity matrix per participant set. The averaging procedure increases the precision of the multidimensional scaling (MDS) by increasing power and reducing error (Li et al., 2020; Fig. 2b). Based on the dissimilarity matrices, the subjective perceptual space of the tones was created with MDS per participant set using the *mds* function of the *smacof* package in *R* (Mair et al., 2022; Fig. 2c). MDS is a widely used descriptive analysis based on similarity judgments. MDS maps relationships between items which can be used to infer the organization of items in a multidimensional space (Hout et al., 2013). In order to create a single STC perceptual space (Fig. 2d, e), we combined the perceptual space of each participant set by applying an affine transformation to match the anchor points using the *computeTransform* function from the *morpho* package (Schlager, 2017). Finally, we drew a smooth curve connecting the dots on the perceptual space with a spline function (Shepard, 1980). *C* was calculated from the area and perimeter of the splined shape (orange line in Fig. 2e) with the following function:

$$\text{Circularity }(C)=4\uppi \times \text{Area}/{\left(\text{Perimeter}\right)}^{2}$$

This function captures the ratio of the area of a polygon with its perimeter. *C* with a value of 1 indicates a perfect circle (see Li et al., 2020, for details). According to simulations by Li et al. (2020), a *C* of 0.9 is close to a perfect circle; we chose a *C* of 0.9 as a threshold for nearly perfect circularity (see Supplementary Fig. S2 for *C* value of equilateral polygons).

#### Statistical testing

Bayesian *t*-tests and ANOVAs were run with JASP 0.16.3 (JASP Team, 2022). Bayes factor (BF) values were interpreted according to Wetzels et al. (2011). BF_10_ values of 1–3 were regarded as anecdotal evidence, 3–10 as substantial evidence, 10–30 as strong evidence, 30–100 as very strong evidence, and BF_10_ values above 100 as decisive evidence in favor of the alternative hypothesis. BF_10_ values of 0.33–1 were regarded as anecdotal evidence, 0.1–0.33 as substantial evidence, 0.03–0.33 as strong evidence, 0.01–0.03 as very strong evidence, and BF_10_ values below 0.01 as decisive evidence in favor of the null hypothesis. Tidyverse (Wickham et al., 2019) and *data.table* R packages were used to preprocess and analyze the data. GGplot2 (Wickham, 2016) was used to create figures.

### Results and interim discussion

#### Similarity ratings

**Fig. 3:**
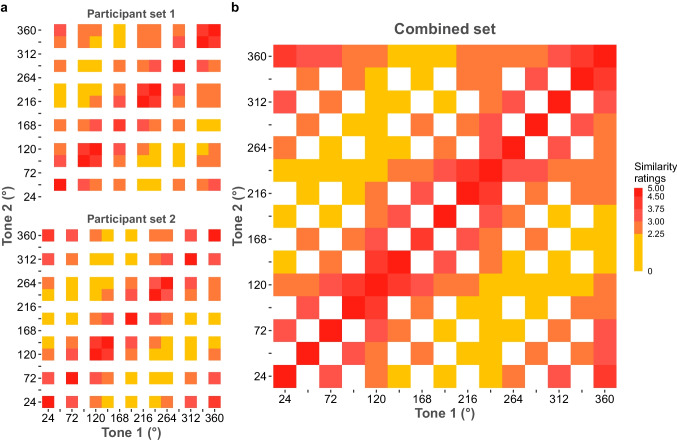
Similarity ratings of tone pairs: **a** per participant group and **b** for the combined participant sets. The combined set consists of tone pair similarity ratings for both participant sets. The *x*- and *y*-axis show the fundamental frequency of tones

Visual inspection of the similarity matrix allowed us to assess whether the STC space wrapped to 360°. Specifically, if the STC space was perceived as circular, pairwise similarity ratings should be determined by angular rather than frequency differences. If the similarities of tone pairs followed a linear pattern, similarity ratings would not recover as the difference in fundamental frequency increased. However, pairwise similarity ratings decreased as the circular distance between tone pairs increased in both participant sets (Fig. 3a) as well as when participant sets were combined (Fig. 3b).

**Fig. 4:**
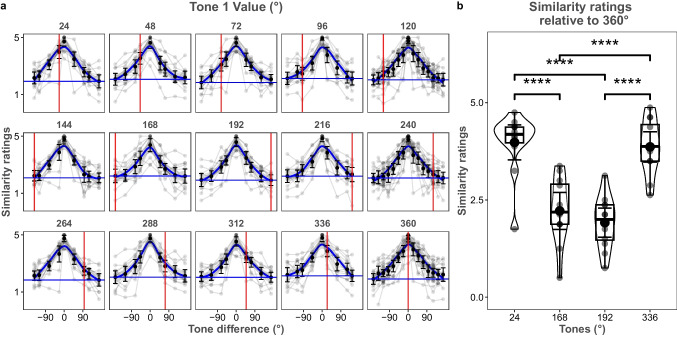
Similarity ratings for each tone and comparison of similarity ratings relative to circle boundary**. a** Similarity ratings as a function of angular distance for each unique tone. The red vertical lines on the panel show 360°, and the blue horizontal lines show the minimum similarity ratings. If perceptual space was not circular, then we would expect abrupt deviations from symmetry near the red vertical line. The blue color shows the fits calculated with the local polynomial regression fitting (loess) function using the formula y ~ x. Error bars represent 95% between-subject confidence intervals. Gray dots show participant data, and gray lines connect each participant’s similarity ratings. **b** Similarity ratings of the closest and furthest tones relative to the circle boundary, 360°. The *x*-axis shows corresponding angles of the tones on the STC space, and the *y*-axis shows similarity ratings. Error bars represent 95% between-subject confidence intervals, and the black dots show the average similarity rating. Transparent dots show each participant’s similarity ratings. Boxplot shows median and quartile values. The violin plot shows the distribution. **** Decisive evidence supporting the difference between conditions

Pairwise similarity ratings for each tone as a function of their angular distance were visually investigated to assess whether the perceptual STC space wrapped around a circle (Fig. 4a). If the STC space wrapped around a circle, there should not be any sharp change in the perceptual similarity scores around the boundary of the circle (360°). Following this prediction, no such sharp change was observed with regard to the similarity ratings of the tones relative to the circular boundary (360°, red vertical lines in Fig. 4a). The minimum pairwise rating for each tone relative to the tone it was compared to was lowest when the angular distance was the greatest (blue horizontal lines in Fig. 4a). This means that as the angular distance between tone pairs increased, their similarity scores decreased. As a final check, we compared the similarity ratings of close (24° and 336°) and far (168° and 192°) tones relative to 360°, with the expectation that those tones with a greater circular distance would show larger differences. This was indeed what was found (Fig. 4b). Bayesian between-subject ANOVA analysis showed decisive evidence that circular distance influences pairwise similarity ratings (*BF*_10_ > 1000; Fig. 4b). Bayesian post hoc tests were conducted to compare the tone similarity ratings. Critically, there was anecdotal evidence against any differences in similarity ratings between 24° and 336°, despite the fact that 24° is the furthest from 360° in unwrapped space (*M*_24°_ = 3.98, *SD*_24°_ = 0.80; *M*_336°_ = 3.87, *SD*_336°_ = 0.69; *BF*_10_ = 0.39). These tests also supported general distance effects. Bayesian post hoc tests showed decisive evidence that the perceptual similarity of tones at 24° and 360° was greater than the perceptual similarity of 360° and 168° or 192° (*M*_168°_ = 2.32, *SD*_168°_ = 0.84; *M*_192°_ = 1.92, *SD*_192°_ = 0.66; *BF*_10_ = 622.28; *BF*_10_ > 1000, respectively). Likewise, decisive evidence was observed when using 336° as the reference point—similarity ratings of tones at 336° and 360° were greater than 360° and 168° or 196° (*BF*_10_ = 591.72; *BF*_10_ > 1000, respectively). All in all, we conclude that circular distance accounts for the Shepard tone’s perceptual similarity.

#### Circularity of the Shepard tone space

**Fig. 5:**
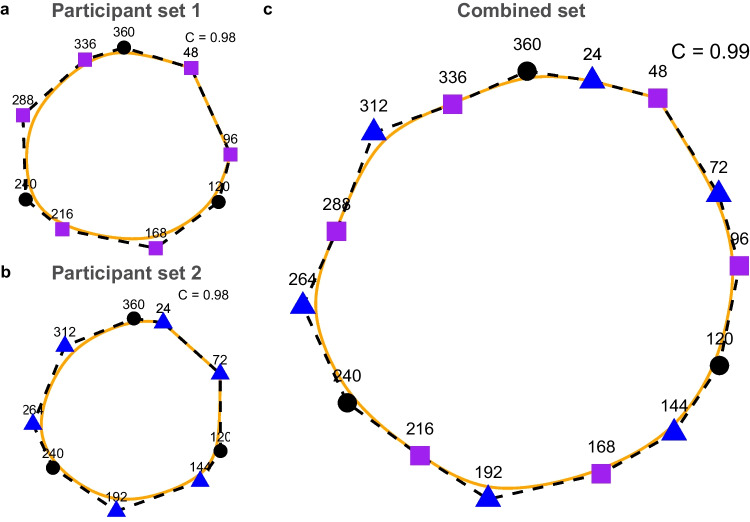
Subjective perceptual space of STC: **a** participant set 1, and **b** participant set 2. **c** Combined subjective perceptual space by using anchor points with affine transformation. Purple squares are the subjective perceptual space of participant set 1, and blue triangles are the subjective space of participant set 2. Black circles are anchor tones. C on the top right side of each panel indicates the circularity value

We mapped the perceptual STC space for each participant set. *C* was 0.98 on the splined area (orange shape in Fig. 5a) and 0.94 on the dashed shape (black dashed polygon in Fig. 5a) in participant set 1. Similar values for *C* were observed for the second participant set, where *C* was estimated as 0.98 on the splined shape (orange shape in Fig. 5b) and 0.95 on the dashed shape (black dashed polygon in Fig. 5b). Combining each participant set’s perceptual space with affine transformation increased *C*. *C* was 0.99 on the splined area of the combined perceptual STC space (orange shape in Fig. 5c) and 0.96 on the dashed area (black dashed polygon in Fig. 5c). *C* with a value of 0.99 can be considered an almost perfect circle, as only an equilateral hexadecagon can achieve the same* C* value (Supplementary Fig. S2)*.* Furthermore, since the *C* value of STC’s perceptual space was above the threshold (*C*_all_ > 0.9), it can be concluded that the perception of the STC space is indeed circular. We also investigated the perceptual space of the STC of each participant separately (Supplementary Fig. S3). Although there were some individual differences in perceptual STC space, the perceptual space approached perfect circularity for most participants (*C* > 0.90 for 16 participants). For the remaining participants, the perceptual space followed a circular path (with *C* < 0.9), except in two cases, for which the perceptual space was not circular. Thus, the overall consistency across participants confirms the prediction that perceptual STC space would form a circle.

We assessed the uniformity of the STC space through a visual inspection for each participant group. This revealed that tone comparisons with small angular distances (24°) were perceived as more similar than tone comparisons with large angular distances (48°). Indeed, the perceptual spaces were uniform for both participant groups (Fig. 5a, b).

## Experiment 2: ART – An auditory delayed estimation paradigm

Continuous reproduction tasks extend our understanding of working memory by utilizing more detailed responses than change detection paradigms or other standard tasks. This allows researchers to formulate and test models that go beyond conceptualizing memory as a discrete, high-threshold system. This model comparison approach has been highly productive, resulting in major advances in the understanding of visual working memory. For example, theoretical models have been developed to separate responses into putative memory states, in order to understand how manipulations such as memory set size separately affect the properties of those states. While there are several competing theories and no consensus (Oberauer, 2021; Schurgin et al., 2020; Van den Berg et al., 2014), a method with detailed generative responses allows for richer model testing than existing approaches.

Prior to a shift toward continuous report tasks, a major thread of research on working memory was to understand the degree to which working memory systems operated in a modality-specific or modality-independent fashion. An unfortunate side effect of the aforementioned progress on visual working memory was a shift away from this important question. As the circular reproduction paradigms have become a standard way of measuring visual working memory, an equivalent paradigm in other domains has become a necessity for a more comprehensive understanding of modality-general working memory functioning. To our knowledge, only one study has used a circular reproduction task to test auditory working memory. Joseph et al. (2015) mapped English vowels on a circle using a two-dimensional formant space, and applied a mixture model to response errors obtained with a novel auditory delayed estimation paradigm. This vowel reproduction methodology utilized to gauge auditory working memory recall precision for phonemes was deemed robust. However, they observed an increase in categorical responses in the face of augmented memory load. It could be that the vowel stimuli space is inherently categorical, which can explain why responses were clustered around prototypic vowels (Iverson & Kuhl, 2000; Kuhl, 1991). Although their stimuli set wraps around a circle, the *C* score of the perceptual space was below the circularity threshold. As a case in point, we retrieved dimension 1 and 2 coordinates in the set size of 1 condition from the perceptual vowel space mapped by multidimensional scaling and calculated the *C* value. The perceptual vowel space did not resemble a perfect circle, as the *C* of the vowel space was 0.81 (i.e., below the threshold of a perfect circle). Overall, since the circular vowel space that was employed by Joseph et al. (2015) produced categorical-bias responses and its perceptual space cannot be considered circular, it does not satisfy the requirements of both uniformity and circularity. Further, outside of experiments conducted by Joseph and colleagues, the paradigm has not generated broad interest in studying auditory working memory.

To overcome the constraints of existing circular auditory reproduction tasks, we created a novel auditory working memory paradigm using an extension of the stimuli set of which the perceptual space was shown to be both uniform and circular in Experiment 1. Furthermore, we tested how the manipulation of set size modulates auditory reproduction performance. Manipulating the number of array items is common in working memory studies (e.g., Bays et al., 2009; Gorgoraptis et al., 2011; Zhang & Luck, 2008), as theoretical models can differ on how this manipulation will affect the pattern of errors. We predicted that remembering two tones would lead to higher error due to higher memory load (Bays et al., 2009; Gorgoraptis et al., 2011; Zhang & Luck, 2008). Further, we modeled our report data using a mixture modeling framework (Bays et al., 2009; Zhang & Luck, 2008; see Supplementary Fig. S6 for signal-detection models) to determine whether these errors arise due to an increase in guessing, less veridical on-target responses, or reporting the wrong item.

The primary goal was to provide a proof-of-concept demonstration of ART for testing formal models of auditory working memory via continuous report data. The hope was that by utilizing equivalent methodological and modeling frameworks for visual and auditory working memory, we could push the field beyond a focus on visual working memory, and toward a theoretical perspective that considers multiple modalities.

### Methods

#### Participants

Although Bayesian tests do not require sample size calculations (Berger & Wolpert, 1988), we aimed to include the data of 18 participants, as the sequential analysis (Schönbrodt et al., 2017) of a pilot study showed that 18 participants would be sufficient for finding the intended effects. In total, 24 participants participated in the experiment, six of whom were excluded from data analysis because their average reproduction error exceeded 60° (indicating low or chance-level performance), which was our a priori allowed maximum error. Participants received 50-dirham vouchers for their participation. The final sample consisted of 18 students (11 female, 7 male) at the New York University Abu Dhabi (mean age = 19.8 years, ranging from 18 to 22 years). All participants reported normal hearing. Consent forms were collected prior to participation, and the study was conducted in accordance with the Declaration of Helsinki (2008).

#### Apparatus

The experiment was run on computers with the Windows 10 operating system, with a screen resolution set to 1280 × 1024, and using MATLAB (The MathWorks, Inc., 2020) with the Psychophysics Toolbox extensions (Brainard, 1997; Pelli, 1997). ATH-M50x over-ear headphones were used for sound playback, and responses were collected with a standard computer mouse.

#### Stimuli

Stimuli were generated using the same method as in Experiment 1. To construct an auditory continuous reproduction task, the full range of the stimulus space (1–360°) was used rather than discrete tones. All other details of the stimuli were identical to Experiment 1.

#### Procedure

**Fig. 6:**
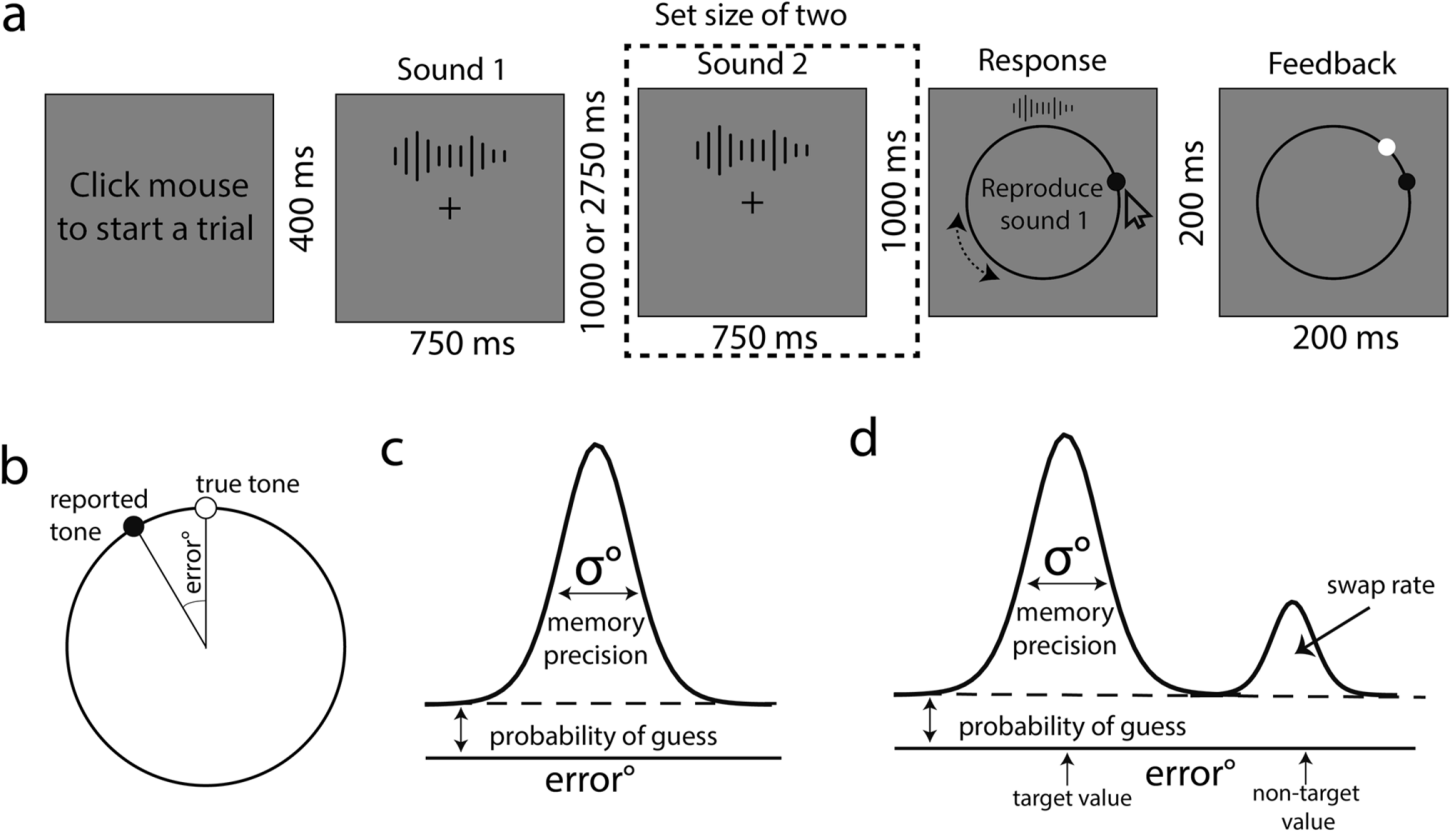
Illustration of the experiment and measures. **a** Illustration of a trial. During presentation of sound 1 and sound 2, there was nothing onscreen besides the fixation; the spectrograms are for illustrative purposes only. Sound 2 (dotted box) only played on set size 2 trials. Otherwise, during this interval only the fixation remained onscreen. **b** Reproduction error. The white dot on the circle shows the presented tone, and the black dot represents the reported tone. The angular difference between the reported and true tones is the reproduction error. Illustration of **c** the standard mixture model and** d** swap model

Following a practice block of eight trials (which participants repeated until five out of the eight trials had less than 45° error), the experiment consisted of 4 blocks of 50 trials each. Every block included an equal number of trials per set size, presented in random order. Trials were self-paced; each trial started with a mouse click, followed by 400 ms of a fixation cross (Fig. 6a). A randomly chosen tone was presented for 750 ms, followed by a 2750-ms retention interval in the condition with a set size of 1. When the set size was 2, another randomly chosen tone was presented for 750 ms after a 1000-ms ISI following the first tone. The final tone was then followed by a 1000-ms retention interval. The total duration from the onset of the first tone until the response was identical between set size conditions, as we considered it important to try to equate (as best as possible) the duration of items in working memory across the two conditions. Participants were instructed to keep their eyes on the fixation cross that was present in the middle of the screen during the trial until the response screen. At the response screen, a circle appeared with a dot positioned at its center. Participants then dragged the dot to the circle using the mouse, locking the dot onto the circle and starting sound playback. After the dot was locked onto the circle, participants could drag the dot to any position on the circle. Changing the position of the dot on the circle, and therefore the angle that corresponded to this position on the circle, simultaneously changed the played-back tone in terms of its fundamental frequency. To prevent participants from remembering the location of the tone rather than the sound information itself, we randomly rotated the reproduction circle each trial. When moving the position of the dot on the circle, sound playback stopped and only started again after leaving the dot in a particular position. After orienting the dot to the position on the circle corresponding to their memory of the presented tone, the participants clicked the mouse to lock in its position. Since it was important for responses to be accurate instead of fast, no response time limitations were implemented. A warning stating “too fast response” appeared on the screen if the response time was less than 750 ms. For set size 2, only the first or second tone (randomly selected) was probed on a given trial. After locking in the position of the dot on the circle, trial-by-trial feedback consisting of the presented tone’s true angle and the participant’s response angle was presented for 400 ms. After the offset of the feedback, the fixation cross remained on the screen for another 400 ms until the end of the trial. The task took approximately 45 min to complete per participant.

#### Design and analysis

There were two conditions: a set size of either 1 or 2, forming a two-factorial within-subject design. Reproduction error was used as the dependent variable and was calculated using the absolute angular difference between true and reported angles (Fig. 6b). Bayesian paired-samples *t*-tests were used for pairwise comparisons. The prior is described by a Cauchy distribution centered around zero with a width parameter of 0.707. The prior corresponds to a probability of 80% that the effect size lies between − 2 and 2 (Gronau et al., 2017).

#### Mixture modeling

According to the standard mixture model, response errors are best represented by a combination of two distinct states of working memory (Zhang & Luck, 2008; Fig. 6c). If a stimulus is stored in working memory, the response value tends to concentrate around the true value of the stimulus, thus producing a von Mises distribution (i.e., a circular normal distribution). The precision of the memorized items is represented by the standard deviation of the von Mises distribution. As the standard deviation of the distribution decreases, responses more accurately reflect the presented items. On the other hand, if working memory fails to store any information regarding the stimulus, the response is randomly distributed and conforms to a uniform distribution. The proportion of the uniform distribution represents the guess rate formed by random responses. The standard mixture model is described by

$$f\left(x;\;g,\sigma\right)=\left(1-g\right)\phi\left(x,k\left(\sigma\right)\right)+g\frac1{2\pi}$$

where *x* is the difference between the response and target angle wrapped between –π and π, *g* is the guess rate, *ϕ* is the von Mises distribution function centered around 0, and *k* is the concentration parameter of the standard deviation (*σ*).

An extension of the standard mixture model was introduced with the swap model (Bays et al., 2009; Fig. 6d). The swap model suggests that true random responses and responding to the wrong item (e.g., reproducing the second item when the first item is probed or vice versa) may both contribute to the guess rate. The swap model is described by

$$f\left(x;\;g,\sigma,\beta\right)=g\frac1{2\pi}+\left(1-g-\beta\right)\phi\left(x,k\left(\sigma\right)\right)+\beta\frac1m\sum \limits_i^m\phi(x_m,k\left(\sigma\right))$$

where *β* is the swap rate, *m* is the number of distractors, and *x*_m_ is the difference between the response and non-target angle wrapped between –π and π.

Using MemToolbox (Suchow et al., 2013) and maximum likelihood estimation, the standard model was fit to each set size condition while the swap model was fit to the set size of 2 condition. We fit the models to the error measures in each set size separately and estimated model parameters per participant.

### Results and interim discussion

#### Error

**Fig. 7:**
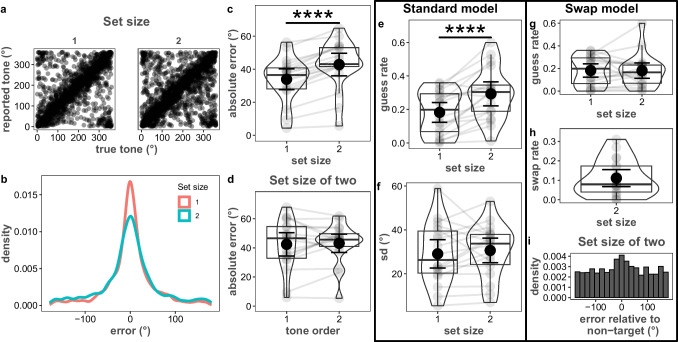
ART performance as a function of set size and presentation order. **a** Plot showing degrees corresponding to the presented tone on the *x*-axis and reported tone on the *y*-axis, separated by set size. **b** Density distribution of the reproduction error as a function of set size. **c**, **d** Average absolute error as a function of set size (**c**) and playback order of the tones with a set size of 2 (**d**). **e**, **f** Guess rate (**e**) and precision (**f**) as estimated by the standard mixture model. **g**, **h** Guess rate (**g**) and swap rate (**h**) as estimated by swap model. **i** Error density distribution relative to non-target tone. Figure conventions follow Fig. 4. **** Decisive evidence against the null hypothesis

We found that performance was higher with one tone than with two. A Bayesian paired *t*-test showed decisive evidence in favor of lower absolute reproduction error with a set size of 1 (33.89°, *SD* = 13.61°) than with a set size of 2 (42.81°, *SD* = 14.70°) (*BF*_10_ > 1000, Fig. 7a–c). Unavoidably, the retention interval of the second tone was shorter than that of the first tone in the set size 2 condition, which can induce a recency effect and shadow the differences between set size conditions. To test whether a recency effect contributed to the current findings, we compared the error between temporal positions (i.e., first and second tone) in the set size 2 condition. There was substantial evidence against the hypothesis of a main effect of recency on the error in the set size 2 condition (*BF*_10_ = 0.25; Fig. 7d). The average error was 42.46° (*SD* = 17.33°) when the first tone was reproduced and 43.15° (*SD* = 13.62°) when the second tone was reproduced. This means that the differences between set size conditions were not affected by a recency effect.

#### Parameter estimations

Bayesian paired-samples *t*-tests showed that differences in reproduction error stemmed from a reduction in on-target responses, not precision. Non-target responses were higher (*BF*_10_ = 235.63; Fig. 7e) with set size 2 (29%, *SD* = 16%) than with set size 1 (18%, *SD* = 13%). Contrarily, there was anecdotal evidence against the effect of set size on the precision of the reproduction errors (*BF*_10_ = 0.31). Average precision was 29.1° (*SD* = 13.93°) with a set size of 1 and 30.65° (*SD* = 12.05°) with a set size of 2 (Fig. 7f). That said, changes were in the direction of larger precision errors for a set size of 2. With a larger increase in set size, we expect that precision differences would indeed be observed.

To determine whether the increase in non-target responses reflected an increase in likely guess responses or was due to participants reporting the wrong item on set size 2, response errors for set size 2 were fit with a swap model (Bays et al., 2009). This model decomposed the 29% non-target responses into 18% guess responses (unrelated to any stimulus) and 11% swap responses. Given that the non-target response rate for set size 1 (20%) was not much different from the guess rate estimated here (*BF*_10_ = 0.24; Fig. 7g), we conclude that the major constraint on performance with two tones in our task is a difficulty in reporting the correct item. That errors in working memory tasks may reflect a difficulty in differentiating amongst stored representations is well documented in studies of visual working memory (Bae & Flombaum, 2013; Bays et al., 2009; Oberauer & Lin, 2017). This suggests that discrimination amongst stored signals is a modality-general constraint on working memory performance. Participants reported the incorrect item in our study more often than is typically observed in visual working memory studies (but see Gorgoraptis et al., 2011). This likely indicates that temporal cues are less effective than spatial cues at cuing to internally held representations (Gorgoraptis et al., 2011). Future research could determine whether spatially separating tones would improve performance by reducing confusability at response.

## Error across blocks

**Fig. 8:**
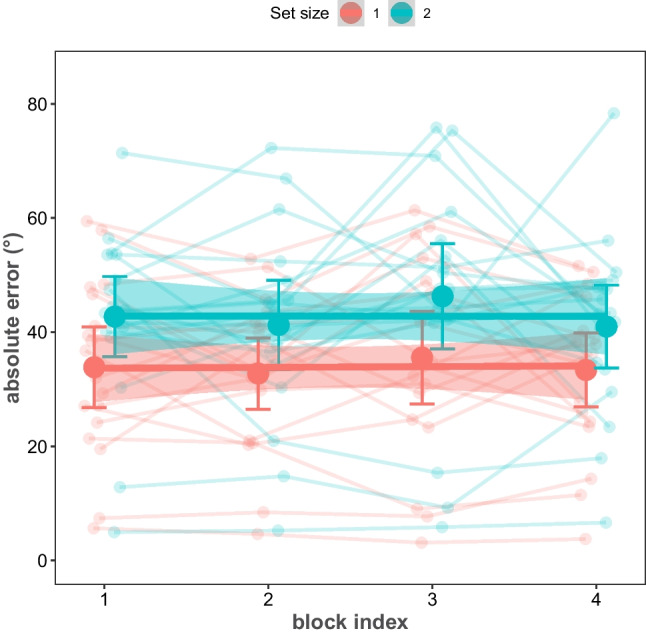
ART errors as a function of set size and block index. Lines are regression fits per set size condition, and shaded areas are the error of the fit. Conventions and other details of the figure follow Fig. 4

In examining performance stability across blocks of trials in ART, a Bayesian within-subject ANOVA was conducted with block number and set size as the independent variables and absolute error in ART as the dependent variable (Fig. 8). For the sake of simplicity, we reported only Bayesian-inclusion factors (*BF*_10-inclusion_) across all-models. The analysis revealed decisive evidence favoring the main effect of set size (*BF*_10-inclusion_ > 1000), substantial evidence against the main effect of block progression (*BF*_10-inclusion_ = 0.19), and strong evidence against the interaction of block progression and set size on absolute errors (*BF*_10-inclusion_ = 0.08). The evidence against the effect of block progression on performance indicates that participants’ performance remained stable throughout the experiment. This suggests that time-on-task factors such as learning, fatigue, or adaptation did not significantly influence the ART errors over the course of the experiment, pointing to the robustness of the task design and the reliability of the auditory reproduction measure in assessing participants’ auditory working memory performance. Furthermore, this finding suggests that if a researcher is only interested in an estimate of performance, and not modeling the reproduction errors, a shorter experiment of about 25 trials per condition would be sufficient, which can decrease the duration of the experiment substantially.

## Experiment 3: Validity of ART

To assess the validity of ART, we correlated ART errors with accuracy in delayed-match-to-sample tasks (DMST) across both auditory and visual domains. DMST, a widely utilized task for evaluating working memory, requires participants to maintain a set of stimuli followed by a delay period. After this delay, participants are asked to determine whether a probe stimulus was part of the initial stimulus set (see Daniel et al., 2016, for a review). Bayesian evidence for a correlation between DMST accuracy and ART errors would provide evidence for convergent validity that ART indeed measures working memory. In addition, under the assumption that there are modality-specific components to working memory tasks (Baddeley, 2003; Fougnie & Marois, 2011; Fougnie et al., 2015), if the correlation between auditory DMST and ART is greater than visual DMST and ART, this finding would provide some evidence that ART draws on auditory working memory. To investigate the validity of the task, participants were administered both auditory and visual versions of the DMST, in addition to the ART, with a set size of 3. We increased the set size to avoid possible ceiling effects in all tasks.

### Methods

#### Participants

In total, 11 participants from Experiment 2 and 13 new participants participated in the experiment. Participants received 50-dirham vouchers for their participation. The final sample consisted of 24 students (10 female, 14 male) at the New York University Abu Dhabi (mean age = 20.9 years, ranging from 18 to 25 years). All participants reported normal hearing, (corrected-to-)normal vision, and normal color vision. Consent forms were collected prior to participation, and the study was conducted in accordance with the Declaration of Helsinki (2008).

#### Apparatus

The experiment was run on computers with the Windows 10 operating system, with a screen resolution set to 1280 × 1024 at 16-bit color depth, and using MATLAB (The MathWorks, Inc., 2020) with the Psychophysics Toolbox extensions (Brainard, 1997; Pelli, 1997) for ART and OpenSesame 3.3.12 (Mathôt et al., 2012) using the Expyriment back-end (Krause & Lindemann, 2014) for auditory and visual DMST. ATH-M50x over-ear headphones were used for sound playback, and responses were collected with a standard computer keyboard and mouse.

#### Stimuli

ART: All details of the stimuli were identical to Experiment 2.

Auditory DMST: Twelve tones were spaced logarithmically using base-2 in a frequency range from 1000 to 2000 Hz. The tone increase was equal to an increase in frequency by a factor of $\sqrt[12]{2}$. Following tone generation, we equalized the root mean square of each of the tones to equalize their amplitude to ensure that low-level features of the sound stimuli were identical. The tones were generated using a sampling frequency of 44,100 Hz.

Visual DMST: Twelve colors with an angular difference of 30° were retrieved from the CIELAB color circle space (Suchow et al., 2013). Colors were presented in a circle with a radius of 0.65° visual angle placed at the center of the screen.

#### Procedure

Three different tasks were used in the experiment: ART, auditory DMST, and visual DMST. The task order was randomized and counterbalanced between participants. The total duration of the experiment was one hour.

ART: The task was identical to Experiment 2 except for the following changes (Fig. 6a). A set size of 3 was used to increase the working memory load to avoid ceiling effects, which may confound the correlation analysis with auditory and visual DMST. There were 80 experimental trials.

**Fig. 9:**
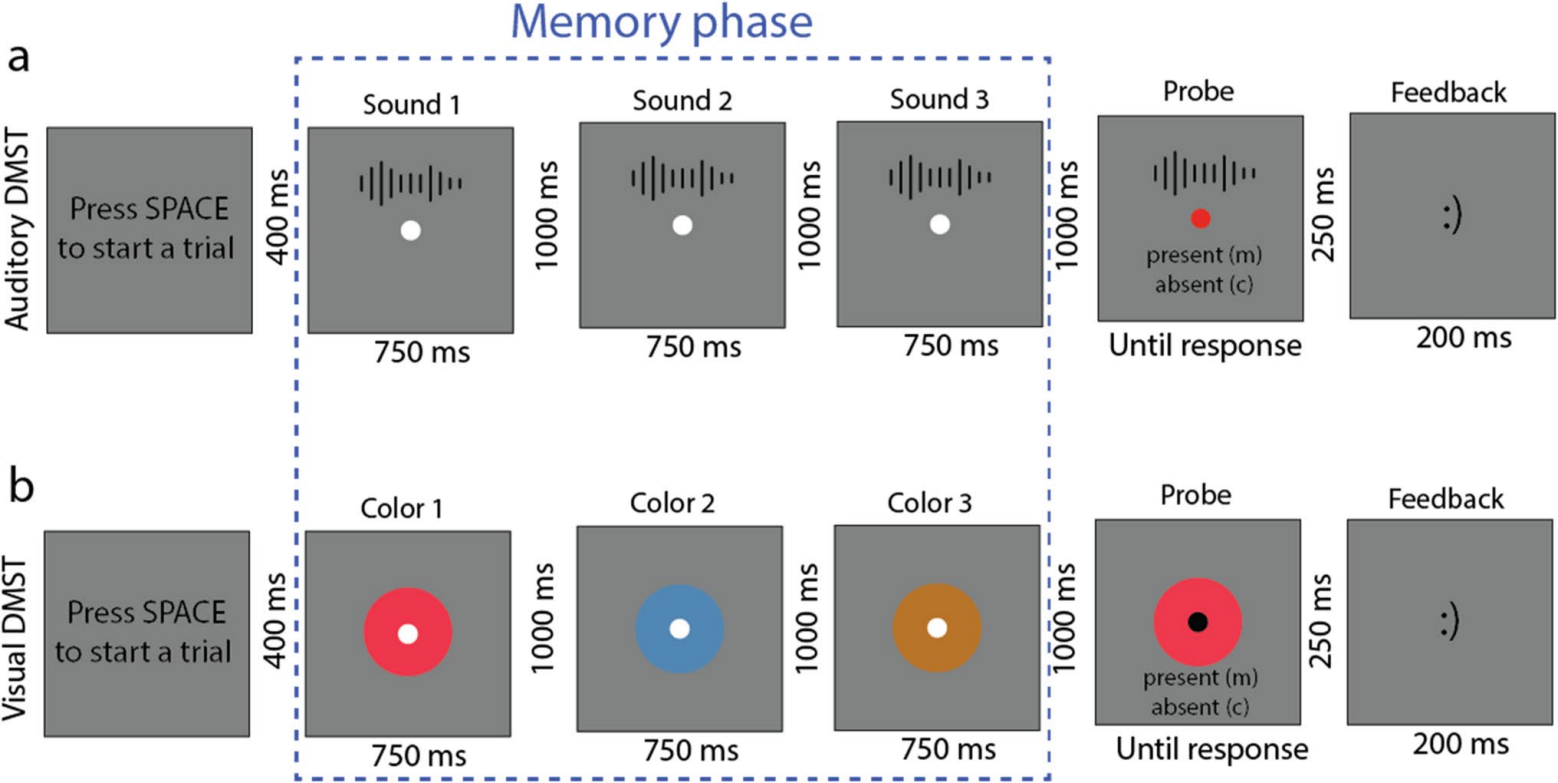
Illustration of the **a** auditory and **b** visual DMST. **a** During tone presentations, there was nothing onscreen besides the fixation; the spectrograms are for illustrative purposes only

DMST: There were eight practice trials in both auditory and visual DMST followed by 48 experimental trials. Three random tones (auditory DMST) or colors (visual DMST) without replacement were chosen for each trial, and each was presented for 750 ms with 1000 ms of ISI between memory items (Fig. 9). There were two types of trials (each equally likely); in the probe-present condition, the probe was included in the sample. In the probe-absent condition, the probe was not included in the sample. In the probe-present condition, the probe was randomly chosen from the first to the third item (equally likely) during the memory phase. There was no response time limitation, and participants were asked to stress accuracy. The fixation color changed after the memory phase to inform participants that the currently presented sound or color was the probe. Participants reported the presence or absence of the probe by pressing “m” or “c” on a standard keyboard. A happy smiley (correct) or unhappy smiley (incorrect) appeared for 200 ms as feedback.

### Results and interim discussion

**Fig. 10:**
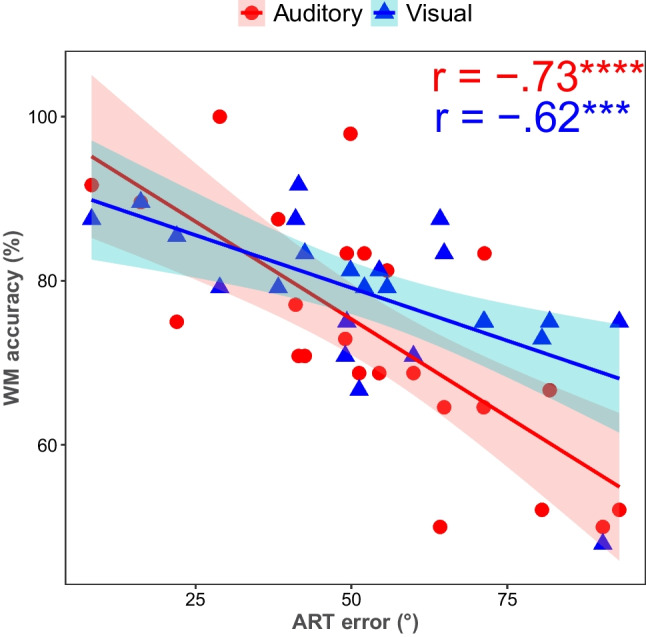
Scatter plot illustrating the correlation between ART errors and DMST accuracy in auditory and visual modalities. The *x*-axis represents the absolute ART error, and the *y*-axis represents the percentage accuracy of the DMST. Red points and the corresponding regression line represent the relationship between ART errors and auditory DMST accuracy, while blue points and the corresponding regression line represent visual DMST accuracy. Each point denotes a single participant’s performance. The shaded areas around the regression lines indicate the 95% confidence intervals for the estimated relationships. *** Very strong evidence and **** decisive evidence against the null hypothesis

Convergent validity of the ART was supported by Bayesian evidence of correlations with both auditory and visual DMST (Fig. 10). Decisive evidence in favor of a strong correlation between ART error and accuracy of the auditory DMST (*r* =  − 0.73, *BF*_10_ = 513.91) was observed, indicating that as the error in ART increased, the working memory accuracy for auditory stimuli decreased. Very strong evidence in favor of a moderate negative correlation (*r* =  − 0.62, *BF*_10_ = 32.09) between ART and accuracy in the visual DMST was observed, though to a lesser extent. The finding of a particularly large correlation between ART and an auditory working memory task suggests that ART draws on similar resources/mechanisms as auditory working memory. Lastly, we investigated test − retest reliability by correlating average ART error in Experiment 2 and Experiment 3. Since the number of participants who participated in both Experiments 2 and 3 was limited, we ran a Bayesian Kendall’s tau rank correlation test. The test results showed strong evidence favoring robust test–retest reliability (*r* = 0.78, *BF*_10_ = 50.92), indicating that ART performance was stable over time, even when the test sessions were separated over weeks.

## General discussion

In recent years, remarkable progress has been made in understanding visual working memory. One of the catalysts of this progress was the implementation of modeling frameworks on continuous reproduction tasks which attempt to reverse-engineer the properties of memory representations that could generate the reproduction errors. While there is still considerable debate over which theoretical model is correct and the degree to which responses arise from separate, and isolatable states (see Oberauer, 2021; Schurgin et al., 2020), there is no debating the influence these models have had on the field of working memory and how this method has shifted the questions that the field is focused on. Unfortunately, the theoretical model framework has narrowed the scope of stimuli used to test working memory. Although there are a few examples of reproduction tasks in the auditory working memory domain, they currently do not mimic commonly used visual tasks due to a lack of circularity and/or uniformity. In this paper, we set out to create a proof-of-concept Auditory Reproduction Task (ART) and test whether the commonly used computational theories of visual working memory are also applicable to auditory working memory. To our knowledge, ART is the first auditory working memory paradigm based on continuous circular reports that utilizes a stimulus space that is free of categorical representations, and is perceptually circular and uniform. In Experiment 1, we first validated the circularity and uniformity of the perceptual space of the Shepard tones used as the auditory stimuli. After verifying this stimulus set, ART was tested within a working memory paradigm with a set size manipulation in Experiment 2. All pre-registered hypotheses were confirmed for both experiments. Error distributions were comparable to those observed in visual continuous reproduction paradigms, with the average error increasing as a function of set size. Furthermore, standard mixture and swap models fit reasonably well on the error distributions obtained through ART (see Supplementary material Fig. S7 for posterior fits per participant). The modeling outcomes suggested that swap errors can explain the worse performance observed with a set size of 2 as compared to a set size of 1, suggesting that misreporting is a modality-general constraint on working memory. In our final study, we found that performance in the ART correlated with delayed-match-to-sample working memory tasks, with a particularly strong correlation between ART and the auditory version of the task, consistent with the argument that ART draws on auditory working memory.

### Perceptual space of Shepard tones

The Shepard tone was created to explore the role of chroma in pitch perception. The perceptual discrimination of Shepard tones follows circular distance rather than linear distance, as was shown with direction discrimination paradigms (Deutsch, 1984; Deutsch et al., 2008; Shepard, 1964; Siedenburg et al., 2023). Extending previous work, we quantified the circularity of the Shepard tone space in Experiment 1. To our knowledge, we are the first to quantify the circularity of the Shepard tones’ perceptual space. We found that it resembled a perfect circle with a circularity score of 0.99 in Experiment 1 (to put this into perspective: achieving a circularity score of this magnitude requires a 19-sided equilateral polygon; Supplementary Fig. S2). Further, by applying multidimensional scaling to each participant, we observed a striking consistency in the circularity of the perceptual space across individuals. Almost all participants’ perceptual spaces of STC adhered closely to a circular configuration (Supplementary Fig. S3). To confirm our findings, we calculated similarity coefficients of target–response pairs from ART following the methodology of Joseph et al. (2015), and assessed their circularity (Supplementary Fig. S4a). The results were even more impressive, as the circularity score was increased to 1.0 when the target and response pairs were binned into 18 equal intervals. Building on this, we revisited the individual perceptual spaces in Experiment 2 and observed improved consistency across participants (Fig. S4b). This enhanced consistency might be attributable to the continuous nature of the target–response pairs used in Experiment 2, in contrast to the subset of tones employed in Experiment 1.

Besides the shape of the perceptual space, an equally important aspect of continuous reproduction paradigms is the uniformity of the tones making up this space. Although the uniformity of the STC space was not tested quantitively, the distances between the individual tones making up the perceptual space for each participant group seemed to be uniformly distributed. As a result, tones with smaller angular distances were perceived as more similar relative to distant tone pairs. Similar to the perceptual space, supplementary analysis (Supplementary Figs. S4 and S5) resembled a perfectly distributed uniform space of the tones when the target and response pairs of Experiment 2 were subjected to multidimensional scaling. As a final check of uniformity, we analyzed the target–response angle pairs for each participant per set size condition in Experiment 2. A visual inspection of these pairs indicated an absence of heavy clustering, suggesting that participants did not exhibit a strong bias toward any specific region of the stimulus space. This further supports the uniform distribution of responses across the circular stimulus space (Supplementary Fig. S5). Last but not least, no categorical representation of the responses was observed, as responses were not clustered around any stimulus value. Therefore, we concluded that the Shepard tones are uniformly distributed on a circle.

### Auditory reproduction task

To date, the majority of auditory working memory tasks have generally utilized discrete stimuli, limiting responses to binary levels (e.g., Fougnie & Marois, 2011; Saults & Cowan, 2007). Although there were a few examples of continuous reproduction paradigms in the auditory modality, they did not become widely adopted as in visual working memory. The reason for this could be the existing paradigms’ limitations or the methodologies’ availability. While both the vowel reproduction (Joseph et al., 2015) and ART tasks have their respective strengths, ART has certain advantages that make it a more suitable option for testing auditory working memory in a continuous circular space. First and most importantly, ART is based on a novel stimulus set that has been validated to be both circular and uniformly distributed. Second, using a novel stimulus set can prevent possible long-term memory interference in working memory paradigms from previous exposure (Blalock, 2015; Jackson & Raymond, 2008), as ART errors are bias-free without categorical responses (e.g., categorical bias in vowel reproduction tasks, Joseph et al., 2015; and color reproduction tasks, Bae et al., 2014). Furthermore, using a novel auditory stimulus set should ensure that neural representations reflect auditory information rather than conceptual or semantic information (see Heinen et al., 2023, for a review). Additionally, ART exhibited a correlation with delayed-match-to-sample working memory tasks, showing a strong association in particular with the auditory version of these tasks, which aligns with the view that ART taps into auditory working memory capabilities. Additionally, ART correlated with delayed-match-to-sample working memory tasks, with a particularly strong correlation between ART and the auditory version of the task, consistent with the argument that ART draws on auditory working memory. Last but not least, ART has proven to be a robust method, demonstrating stable performance across trials and sessions. This stability and specificity suggest that ART could be a useful tool for exploring the differences between auditory and visual memory processing in cognitive research.

### Auditory and visual working memory reproduction compared

One of the central questions motivating ART is whether the theoretical models developed in the visual working memory literature reflect domain-general or domain-specific constraints. While our Experiment 2 reflects only a start in answering this question, the findings do suggest some common constraints on working memory, as well as some possible differences between the auditory and visual domains. Participants’ performance on ART was broadly similar to that observed in the visual working memory literature. As in visual working memory paradigms, errors increased for set size 2 relative to set size 1. When the source of error was examined via a mixture modeling analysis that separates changes in response error as arising due to a loss of precision, an increase in random responses, or swap errors, we found a significant source of response errors at set size 2, but no difference in memory precision or random responses. Consistent with this, several studies in the visual working memory literature highlight that differentiating between stored information can result in correspondence errors or difficulty in reporting the correct stimulus (Bae & Flombaum, 2013; Bays et al., 2009; Oberauer & Lin, 2017). That similar errors arise for visual and auditory working memory suggests that this may be a shared constraint on performance.

In addition to highlighting some similarities, the present work also suggests some potential areas of divergence between visual and auditory reproduction tasks. The average error (and precision parameter) was relatively high compared to average errors reported in the visual working memory literature (e.g., Gorgoraptis et al., 2011; Karabay et al., 2022; Zhang & Luck, 2008). This difference has several potential explanations, including a lower capacity of auditory working memory (Fougnie & Marois, 2011; Lehnert & Zimmer, 2006), a lower representational quality of auditory memory (Gloede & Gregg, 2019), differences in the similarity/discriminability of the stimulus space (see Schurgin et al., 2020), greater confusability amongst auditory items due to the lack of spatial location as an efficient marker, or differences in responses due to constant and changing auditory input. Further, visual working memory studies typically find decreased memory precision with larger set size (greater standard deviation of error distributions), even if the increase in set size is still lower than the presumed item capacity (e.g., Zhang & Luck, 2008; but see Bae & Flombaum, 2013). Our estimates of memory precision for set size 1 and 2 were equivalent. Does this mean that, unlike visual working memory, auditory working memory has no difference in representational quality? There are reasons to be skeptical. Precision differences in visual working memory tend to be relatively small, and our precision estimates were relatively imprecise, which would lead to noisier estimates. Future research is necessary to address this question. Finally, our swap error estimates were larger at set size 2 than is found for most studies on visual working memory. However, we believe this reflects the use of a temporal order cue rather than differences in stimulus modality. Indeed, even larger swap rates were found within the visual domain when temporal order was used as the cue (Gorgoraptis et al., 2011). Practically, researchers will need to be careful to minimize confusability when increasing set size beyond two items.

Critically, the goal of analyzing our data was not to adjudicate between competing theoretical models, but to show that auditory working memory can be part of this conversation. Therefore, we were focused on interpreting our findings in terms of the more dominant models in the literature. Notably, more recent models have rejected the idea of separate, putative states. According to the target confusability competition model, error distributions can be explained by a single memory strength parameter (Schurgin et al., 2020). When applied to our results, the memory strength parameter decreased at set size 2 compared to set size 1 (Supplementary Fig. S6). Regardless of which theoretical model one prefers, the ability to generalize models into another sensory domain will be of value.

### Future directions

ART generates several testable research questions. For example, there are different types of distortions that have been identified in visual working memory, such as ensemble integrations, central tendency or repulsion, and attractions (Brady & Alvarez, 2011; Chunharas et al., 2022; Hollingworth, 1910). We do not know, however, whether these distortions are specific to visual working memory, or whether they represent a modality-independent function of working memory. If these working memory distortions are not specific to vision alone, it would be expected that similar distortions exist for auditory working memory.

## Conclusion

We aimed to fill a gap in the literature on auditory continuous reproduction tasks with a novel methodology: ART. After confirming the circularity and uniformity of the Shepard tone space, we demonstrated the applicability of this task to the common theoretical working memory models in the literature. We think that ART has potential to benefit the field by allowing theoretical and computational working memory models built on visual continuous reproduction tasks to be tested in auditory modality, enabling a shift in the working memory field from the visual modality to a more general modality-independent understanding of working memory. In sum, ART provides a promising avenue for future research in this field and has the potential to contribute significantly to our understanding of cognitive processes beyond working memory.

## Supplementary information

Below is the link to the electronic supplementary material.

Supplementary file1 (PDF 856 KB)

## Data Availability

All data including experimental tasks are publicly accessible on the Open Science Framework with the identifier WF5UV (osf.io/wf5uv/).

## Code availability

Analysis scripts are publicly accessible on the Open Science Framework with the identifier WF5UV (osf.io/wf5uv/).
