# Supplementary material for: Introducing ART: A new method for testing auditory memory with circular reproduction tasks
Source: Behav Res Methods. 2024 Sep 9;56(8):8330–48. doi: 10.3758/s13428-024-02477-2 (PMC11525316; doi:10.3758/s13428-024-02477-2)
Supplement: Supplementary file 1 — Supplementary file1 (PDF 856 KB) [file 13428_2024_2477_MOESM1_ESM.pdf]

Supplementary: Introducing ART: a new method of testing auditory memory with circular reproduction tasks

1. Are similarity responses continuous?

Figure S1 shows the frequency distribution of the similarity ratings. Participants did not choose minimum and maximum similarity ratings as often; instead, the similarity ratings resemble a normal distribution.

Figure S1. *Frequency distribution of similarity ratings*

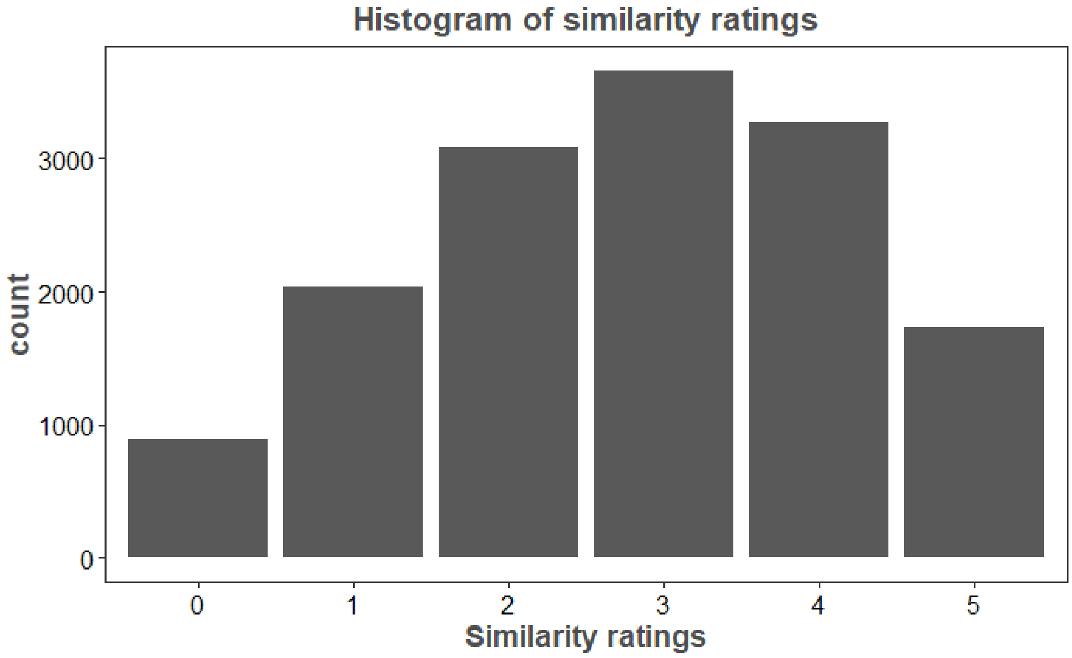

2. Circularity value of equilateral polygons

We assessed how the number of sides of a polygon influences the circularity value (*C;* Figure S2). We computed the *C* for equilateral polygons with the number of sides ranging from 3 to 20. An equilateral hexagon passes the circularity threshold (.90). It should be noted that this analysis is conservative as we calculated *C* for regular polygons.

Figure S2. Circularity values of equilateral polygons

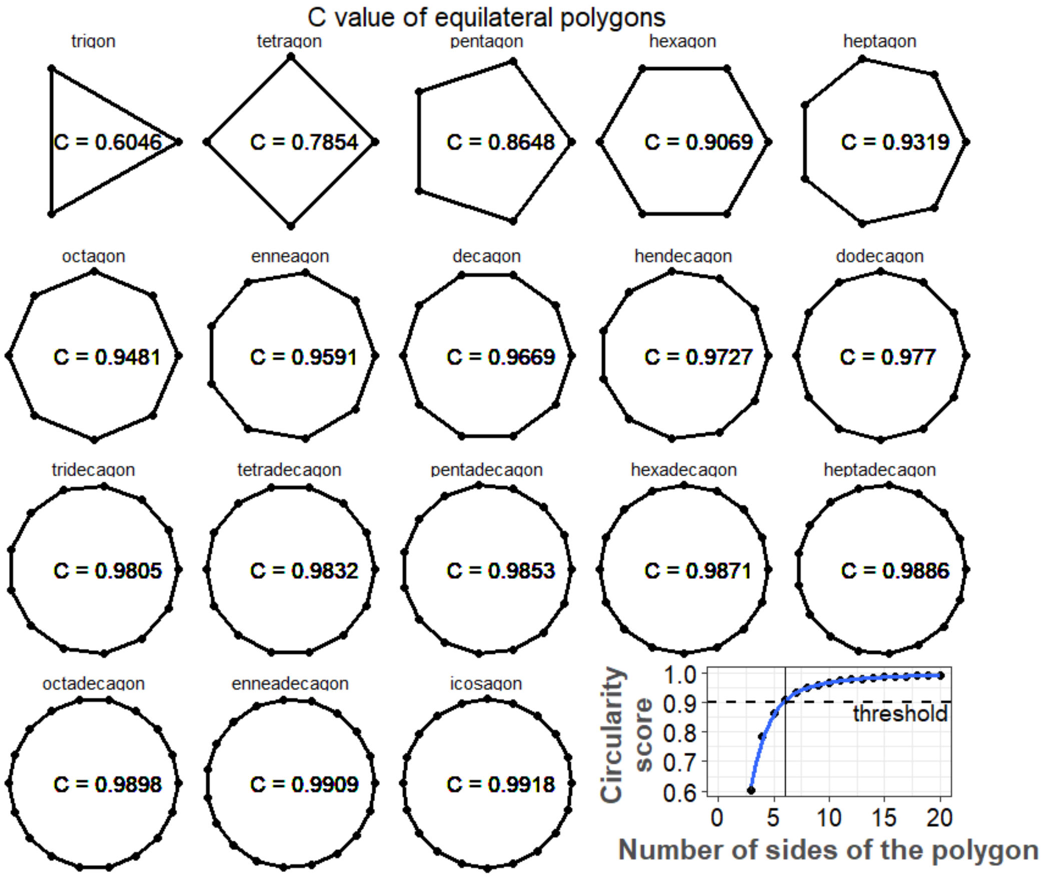

3. **Individual differences in the perceptual STC space**

We calculated the *C* value of the perceptual STC of each participant to assess whether there were any individual differences in perceptual STC space. Indeed, the perceptual STC space of 16 participants was above the threshold (*C* > .9; Figure S3). Also, the perceptual STC space of the other participants resembled a circle. However, their *C* value was below the threshold (Participants 2, 4, 12, 15, 17, 23). Only two participants` perceptual STC space did not form a circular shape (Participants 6 and 25). A deeper look at the similarity ratings of participants 6 and 25 revealed that they did not use the similarity ratings continuously. It could be that their way of responding to the similarity rating scale, rather than the perceptual STC space, would caused the non-circularity of their perceptual STC space.

Figure S3. *Representational STC space of each participant*

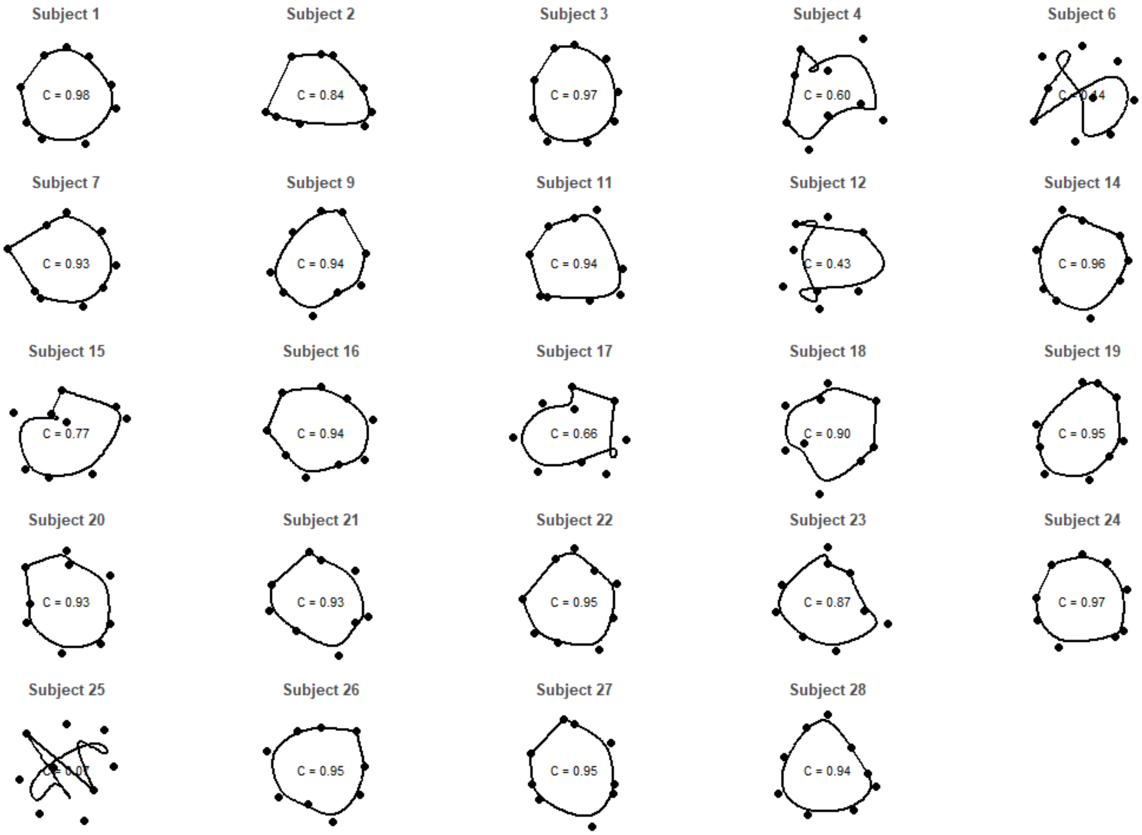

4. The circularity of response error in the reproduction task

We generated similarity coefficients from the target-response angle by retrieving the relative ratio of every possible bin. First, target and response values were binned into 18 discrete angle groups. Second, for each target bin, the proportion of response bins was considered a similarity coefficient. For example, for bin 1 (0°-20°), we calculated the proportion of responses in the same bin and all other bins, which was considered the similarity coefficient. This procedure was done for all target bins. We subtracted similarity coefficients from 1 to generate a 20 by 20 matrix with dissimilarity values. We then followed the same protocol as in the assessing circularity section of Experiment 1 to calculate the *C* value of the tone reproduction task. Strikingly, we observed a perfect circle (orange smoothed line C = 1.0, black dashed-line = .98 Figure S4a) with a perfectly uniform distribution of tones forming a circle. Testing with different bins did not influence the representational space's circularity and uniformity unless there were only a few trials in each bin. Furthermore, we checked if this pattern was similar at the individual level.

For this analysis, we decreased the number of bins to 12 because there were fewer data points at the individual level. Subject-level analysis showed that the *C* value of 14 out of 18 participants' perceptual space was above the threshold (*C* > .9; Figure S4b). The perceptual space of the rest of the participants also resembled a circle (except Participant 4). With visual inspection, we observed that the angular difference of tones in most participants' perceptual space was uniform.

Figure S4. *Representational space of tones in the ART*

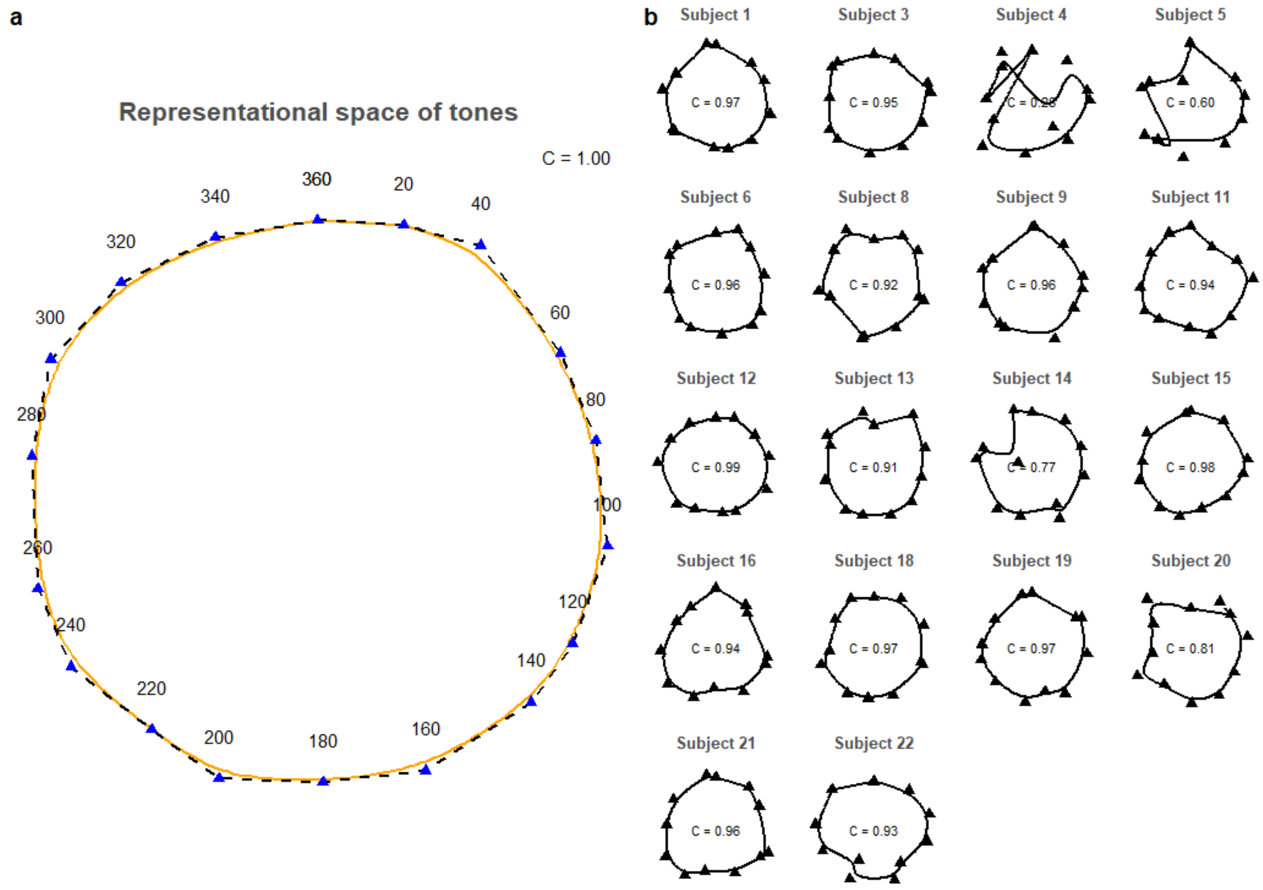

5. Distribution of responses relative to target values

If the uniformity and circularity assumptions of the tones are met in ART, one would expect that individual responses would form a uniform distribution that wraps around the circle. Indeed, when plotting response values against target values for each set size condition in Experiment 2, per participant, it appears that the perceptual space for each individual is circular, as evidenced by the clustering of responses at the extremes of the off-diagonal. Furthermore, there is no substantial bias towards any specific region of the stimulus space; responses seem to be uniformly distributed across the

entire space. Overall, these participant-level target-response values further support the circularity and uniformity of the perceptual space in the ART.

Figure S5. *Scatterplots of target tones and reported tones per participant across size conditions in Experiment 2. Each panel represents data from one participant.*

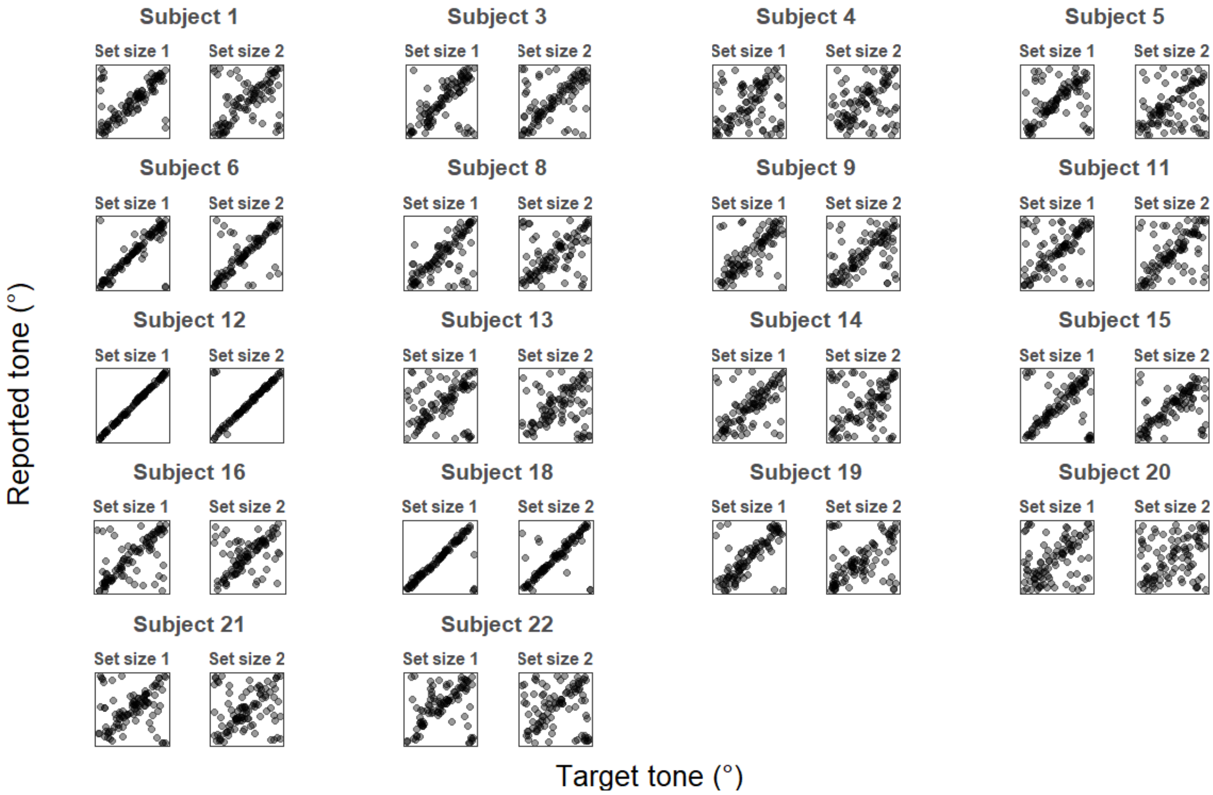

6. Fitting signal-detection working memory models to ART errors

Schurgin and colleagues (2020) introduced a novel measurement model for continuous reproduction that differs from existing models. The new model, called the target confusability competition (TCC) model, treats the continuous reproduction task as a 360-alternative forced choice task with 360° on the circular response scale as possible response alternatives. The TCC model encodes an array and increases the activation of each feature in the array by a certain amount. This increase in activation generalizes to neighboring features based on their similarity. The model's psychophysical scaling function maps the distance between features on the circular scale to their similarity with noise. The response option with the highest familiarity signal is selected as the response. The TCC model has only one free parameter (d'), which can account for variations in the response distribution across different experimental conditions, such as memory set size, presentation duration, and retention interval. Using maximum

likelihood estimation, we fitted the uncorrelated TCC model to errors as a function of set size. We observed substantial evidence favoring an effect of set size on the memory strength of the TCC model (BF_{10} > 1000; Figure S6). The average memory strength was 2.07 (*SD* = .98) in the set size of 1 and decreased to 1.68 (*SD* = .91) in the set size of two.

Figure S6. *TCC parameter memory strength across set size conditions*

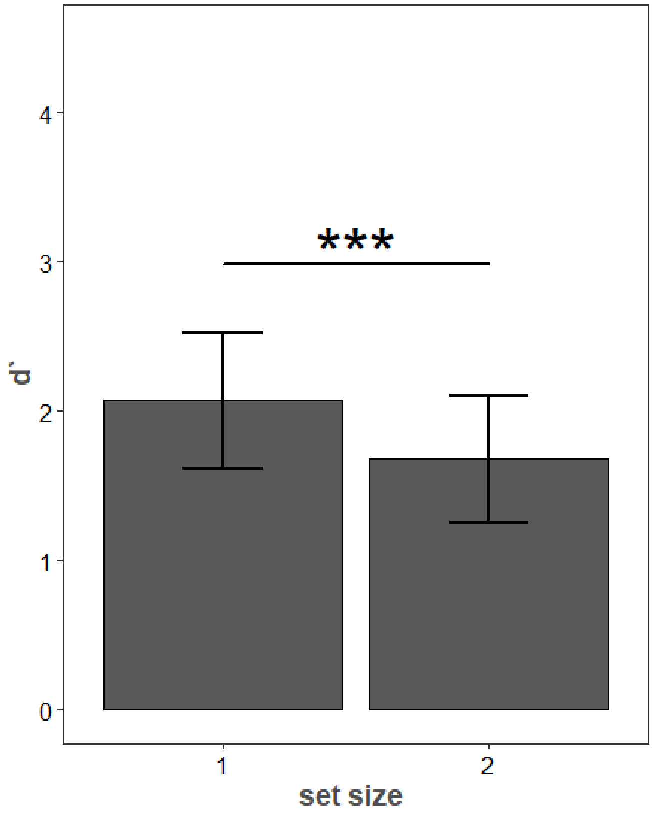

7. **Posterior predictive checks of standard mixture and swap models as a function of set size for each participant**

A visual inspection was carried out for model fits on error distributions per participant (see Figure S7 for individual fits). ART errors and posterior fits with predicted parameters of the standard mixture model were coherent. ART errors were clustered around the target value (precision) and the long tail of the distribution resembles a uniform pattern (guess rate).

Figure S7. *Posterior predictive checks of model fit as a function of set size per participant. Std means standard mixture model, SW means swap model, N represents the set size, and S means participant number.*

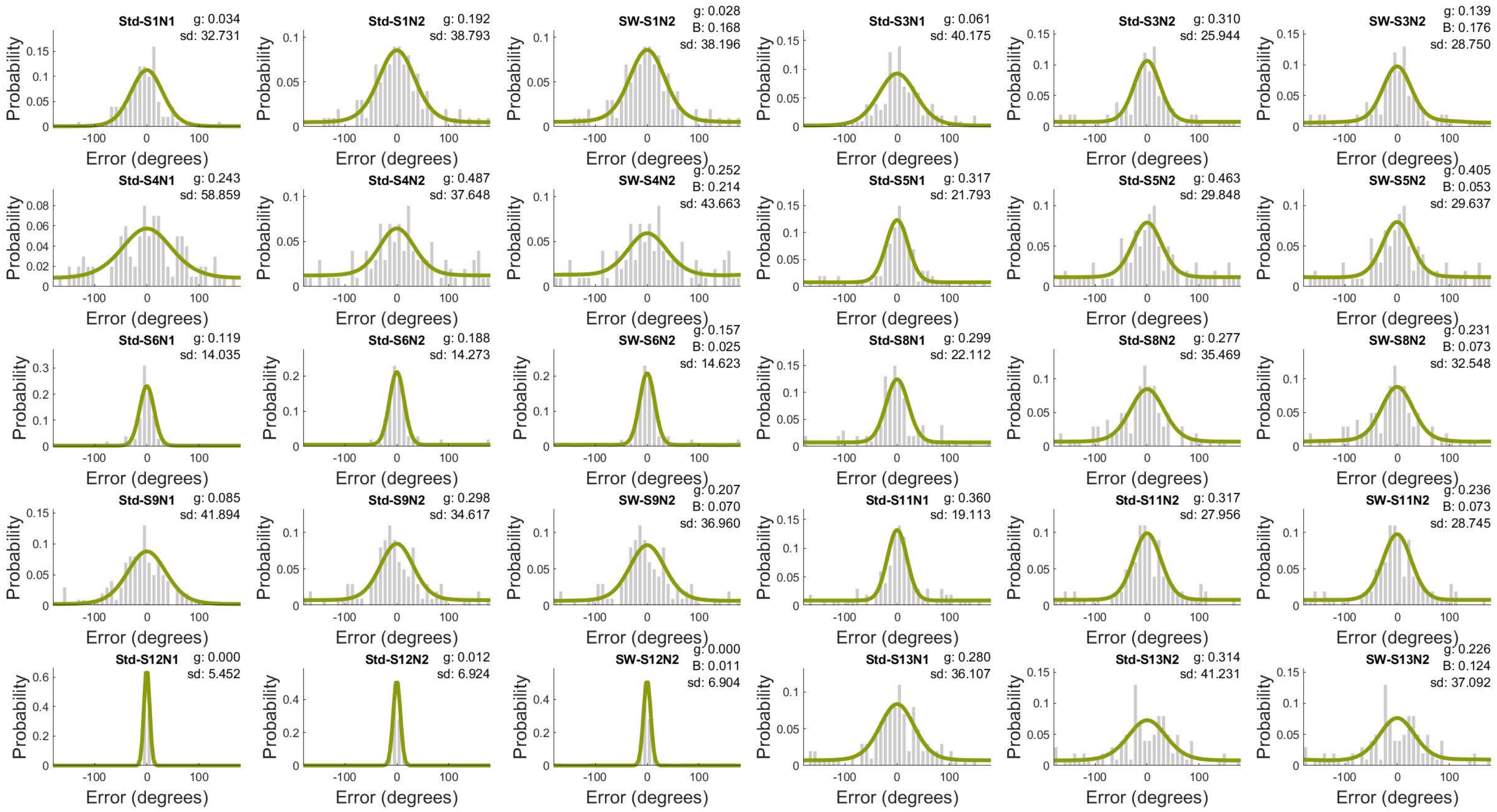
Std-S1N1 g: 0.034 sd: 32.731
Std-S1N2 g: 0.192 sd: 38.793
SW-S1N2 g: 0.028 B: 0.168 sd: 38.196
Std-S3N1 g: 0.061 sd: 40.175
Std-S3N2 g: 0.310 sd: 25.944
SW-S3N2 g: 0.139 B: 0.176 sd: 28.750
Std-S4N1 g: 0.243 sd: 58.859
Std-S4N2 g: 0.487 sd: 37.648
SW-S4N2 g: 0.252 B: 0.214 sd: 43.663
Std-S5N1 g: 0.317 sd: 21.793
Std-S5N2 g: 0.463 sd: 29.848
SW-S5N2 g: 0.405 B: 0.053 sd: 29.637
Std-S6N1 g: 0.119 sd: 14.035
Std-S6N2 g: 0.188 sd: 14.273
SW-S6N2 g: 0.157 B: 0.025 sd: 14.623
Std-S8N1 g: 0.299 sd: 22.112
Std-S8N2 g: 0.277 sd: 35.469
SW-S8N2 g: 0.231 B: 0.073 sd: 32.548
Std-S9N1 g: 0.085 sd: 41.894
Std-S9N2 g: 0.298 sd: 34.617
SW-S9N2 g: 0.207 B: 0.070 sd: 36.960
Std-S11N1 g: 0.360 sd: 19.113
Std-S11N2 g: 0.317 sd: 27.956
SW-S11N2 g: 0.236 B: 0.073 sd: 28.745
Std-S12N1 g: 0.000 sd: 5.452
Std-S12N2 g: 0.012 sd: 6.924
SW-S12N2 g: 0.000 B: 0.011 sd: 6.904
Std-S13N1 g: 0.280 sd: 36.107
Std-S13N2 g: 0.314 sd: 41.231
SW-S13N2 g: 0.226 B: 0.124 sd: 37.092
Probability
Error (degrees)
-100
0
100

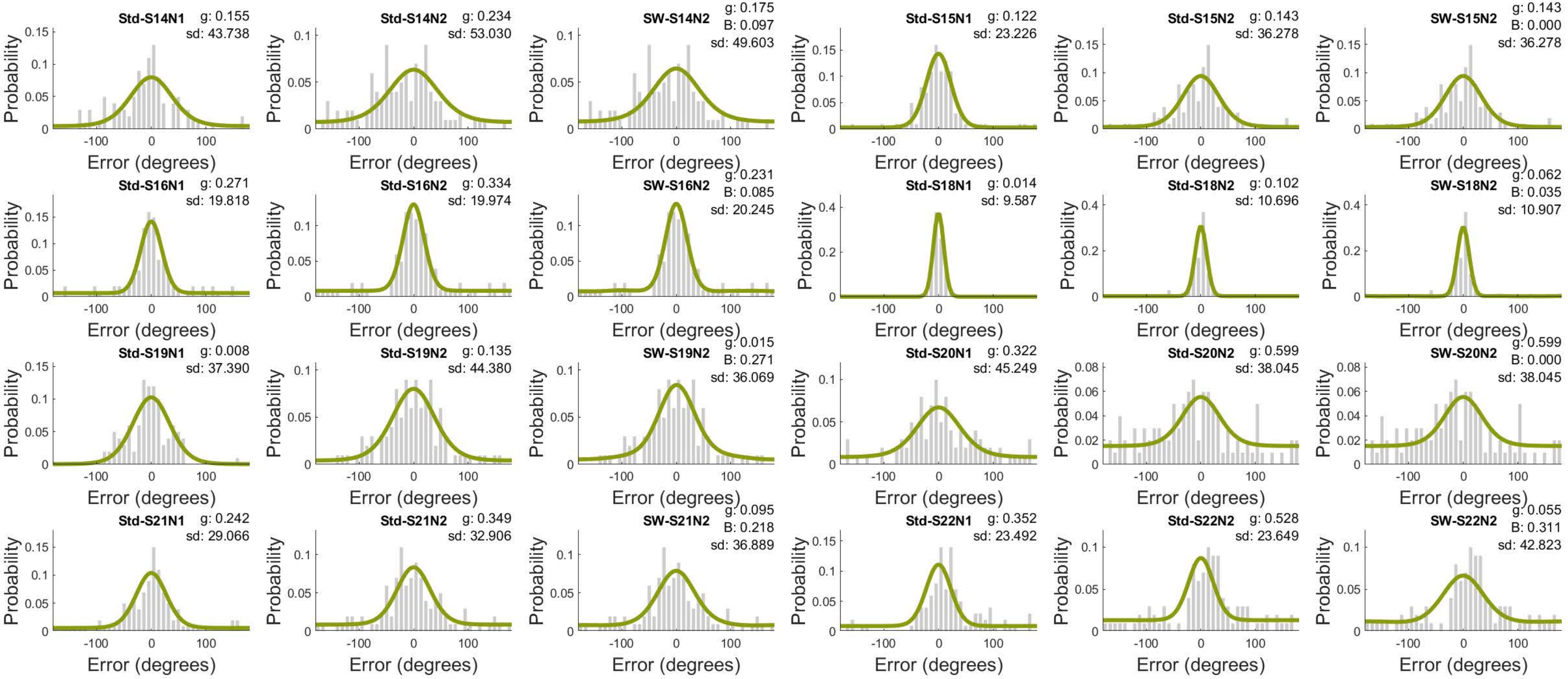

Std-S14N1 g: 0.155 sd: 43.738
Std-S14N2 g: 0.234 sd: 53.030
SW-S14N2 g: 0.175 B: 0.097 sd: 49.603
Std-S15N1 g: 0.122 sd: 23.226
Std-S15N2 g: 0.143 sd: 36.278
SW-S15N2 g: 0.143 B: 0.000 sd: 36.278
Std-S16N1 g: 0.271 sd: 19.818
Std-S16N2 g: 0.334 sd: 19.974
SW-S16N2 g: 0.231 B: 0.085 sd: 20.245
Std-S18N1 g: 0.014 sd: 9.587
Std-S18N2 g: 0.102 sd: 10.696
SW-S18N2 g: 0.062 B: 0.035 sd: 10.907
Std-S19N1 g: 0.008 sd: 37.390
Std-S19N2 g: 0.135 sd: 44.380
SW-S19N2 g: 0.015 B: 0.271 sd: 36.069
Std-S20N1 g: 0.322 sd: 45.249
Std-S20N2 g: 0.599 sd: 38.045
SW-S20N2 g: 0.599 B: 0.000 sd: 38.045
Std-S21N1 g: 0.242 sd: 29.066
Std-S21N2 g: 0.349 sd: 32.906
SW-S21N2 g: 0.095 B: 0.218 sd: 36.889
Std-S22N1 g: 0.352 sd: 23.492
Std-S22N2 g: 0.528 sd: 23.649
SW-S22N2 g: 0.055 B: 0.311 sd: 42.823
Probability
Error (degrees)
